# Functional/dissociative seizures in a single women’s rehabilitation center in Addis Ababa, Ethiopia: a single-center case series and thematic analysis of neuropsychiatric presentation, cultural meaning, and structural barriers to care

**DOI:** 10.3389/fpsyt.2026.1903222

**Published:** 2026-07-31

**Authors:** Yared Alemu, Yoeal Kahsay, Fikirte Weldearegay, Lidya Hailu, Tsion Fekadu, Patrick Ohiomoba, Selam Negussie

**Affiliations:** 1 AYA-Innovation, Addis, Ababa, Ethiopia; 2 Unity University, Addis, Ababa, Ethiopia

**Keywords:** cultural psychiatry, dissociation, Ethiopia, functional neurological disorder, functional/dissociative seizures, gender-based violence, psychiatric rehabilitation, psychogenic non-epileptic seizures

## Abstract

**Background:**

Functional/dissociative seizures (FDS; previously termed psychogenic non-epileptic seizures, PNES) are disabling functional neurological events associated with trauma, dissociation, psychiatric comorbidity, impaired functioning, and increased mortality. In sub-Saharan Africa, limited access to video-electroencephalography (video-EEG), limited specialist services, and culturally embedded explanatory models complicate recognition and treatment. To our knowledge, there are no published Ethiopian case-based studies of FDS employing structured clinical formulation and thematic analysis.

**Methods:**

We present six young women, aged 18–24 years (median 20), identified during routine care in a residential rehabilitation setting in Addis Ababa with clinically suspected FDS. Because video-EEG was unavailable, cases were formulated using positive seizure-like episode features, psychiatric assessment, trauma history, cultural attributions, applying the staged diagnostic framework proposed by LaFrance et al. Treatment response was recorded as a clinical outcome and was not used as diagnostic evidence. The de-identified case narratives were analyzed using framework-based thematic analysis informed by Braun and Clarke, organized around a multidisciplinary model spanning neurobiology, trauma and attachment, dissociation, cultural psychiatry, and structural violence.

**Results:**

Episodes exhibited features consistent with clinically suspected FDS: prolonged duration, irregular or asynchronous motor activity, forced eye closure or side-to-side head movements in some cases, preserved or fluctuating awareness, sensory prodrome in one case, limited recall, absence of postictal confusion, and emotional or interpersonal triggers. Psychiatric features included depression, anxiety, self-harm, substance use, khat-related arousal, nightmares, and suicidality. Trauma and attachment-related vulnerabilities included parental abandonment, maternal rejection, sexual abuse, family-arranged child marriage, homelessness, restricted socialization, estrangement, and separation from a child. Five overarching themes were identified: (1) neurological episode features; (2) betrayal and attachment disruption; (3) dissociation and functional shutdown; (4) psychiatric risk and comorbidity; and (5) culture, gender, and systemic invisibility.

**Conclusion:**

Clinically suspected FDS was identified in young women at a single Ethiopian rehabilitation center and may be under-recognized in comparable settings. These single-center observations are hypothesis-generating and require confirmation in larger studies with electrophysiological support. In settings where video-EEG is unavailable, culturally adapted screening, trauma-informed assessment, suicide risk evaluation, treatment of psychiatric comorbidity, and clear referral pathways are urgently needed.

## 1 Introduction

Functional/dissociative seizures (FDS), also known as psychogenic non-epileptic seizures (PNES) are paroxysmal events that resemble epileptic seizures but occur without the epileptiform electrical activity that characterizes epilepsy. They are classified within Functional Neurological Disorder and are recognized in DSM-5-TR as Functional Neurological Symptom Disorder. FDS is a common and disabling condition that is frequently misdiagnosed and associated with trauma exposure, dissociation, psychiatric comorbidity, functional impairment, and increased mortality (1–3). Across international multicenter studies, FDS predominantly affects women with onset in early adulthood, though such cross-cultural data remain limited in number and quality (4).

Recognition of FDS in Ethiopia is constrained by limited access to video-EEG, a shortage of specialist neurological services, under-recognition of functional neurological disorders, and culturally embedded explanatory models such as Zar (a possession-trance idiom of distress in which intrusive spirits are appeased and managed rather than expelled), Buda (the “evil eye,” a power attributed to certain individuals believed to cause illness or misfortune through envy), Azurit (a locally recognized spirit affliction), evil eye (illness or misfortune attributed to a malicious or envious gaze), spirit possession (attribution of seizure-like or altered states to an inhabiting spirit), holy water (tsebel; sanctified water central to Ethiopian Orthodox ritual healing), and exorcism (ritual expulsion of an afflicting spirit). These frameworks may reduce shame and provide meaning, yet they can also delay biomedical care when they become the exclusive pathway to care (5, 6). The global FDS literature is heavily skewed toward high-income settings, with only 0.9% of published research originating from Africa, leaving the disorder’s presentation on the continent largely undescribed (7).

This manuscript describes six young women with clinically suspected FDS identified in a residential rehabilitation setting in Addis Ababa. It examines their neuropsychiatric presentation, trauma and attachment histories, cultural meanings, gendered and socioeconomic vulnerabilities, treatment pathways, short-term outcomes, and cross-case themes. The aim is to make clinically suspected FDS visible in an Ethiopian rehabilitation setting and to inform culturally responsive social psychiatry and psychiatric rehabilitation. To make the article self-contained, Section 2 sets out the conceptual framework used throughout; Section 3 describes the setting, diagnostic logic, and analytic approach; Sections 4 and 5 present the cases and thematic analysis; and Sections 6–8 discuss implications, limitations, and conclusions.

## 2 Conceptual framework

**Figure 1:**
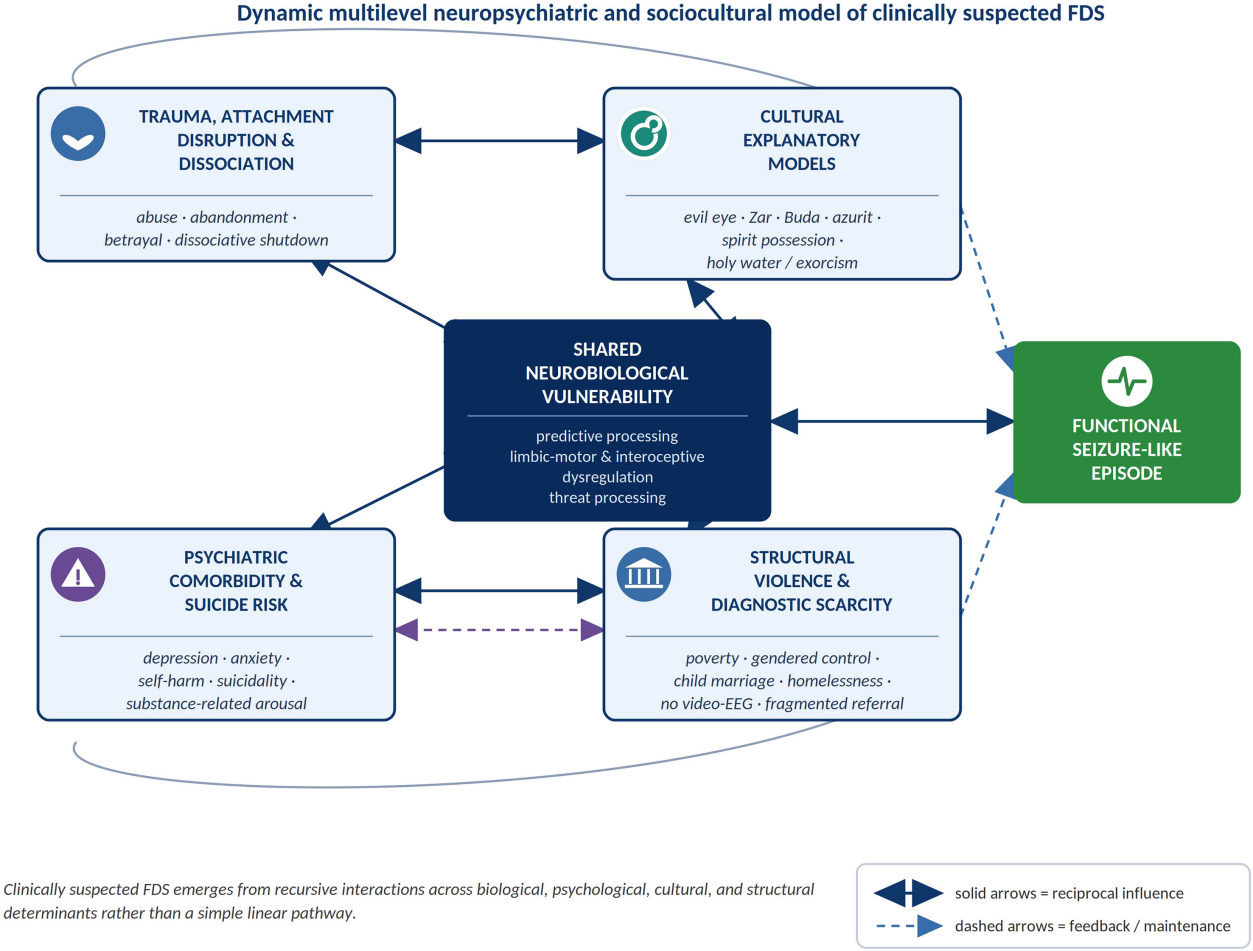
Integrated neuropsychiatric and sociocultural model of clinically suspected FDS in a low-resource rehabilitation setting. Nested determinants converge on a shared neurobiological substrate; functional seizure-like episodes emerge when affective and relational threat exceed regulatory capacity.

The interpretation of the cases in this article draws on five interlocking bodies of evidence. Because no single discipline adequately explains FDS in this population, we integrate them into the layered model summarized in **Figure 1**, in which a shared neurobiological substrate is shaped by trauma, dissociation, psychiatric comorbidity, cultural meaning, and structural conditions. Each formulation in Section 4 refers back to the relevant subsection below.

### 2.1 Neurobiology and predictive processing

Contemporary models conceptualize FDS as a brain-based disorder of nervous system functioning rather than a form of feigned or malingering (8). Recent predictive-processing models propose that prior experiences, including traumatic experiences, may shape expectations and perceptual inferences that influence the interpretation of bodily sensations, emotions, and threat-related signals (9). Neuroimaging studies have reported structural changes on brain MRI (10) as well as altered functional activity and connectivity across limbic, motor, and self-referential networks (11) in patients with FDS, collectively suggesting dysregulated emotion processing, interoception, and motor control. Within this framework, FDS episodes may be understood as involuntary motor manifestations that emerge when emotional or threat-related arousal overwhelms the brain’s capacity for cognitive control, manifesting as physical symptoms.

### 2.2 Mortality, suicide, and psychiatric risk

FDS carries significant clinical risk and should not be regarded as a benign condition. Standardized mortality is elevated, approaching that of severe epilepsy, with excess deaths concentrated in younger adults (2). Psychiatric comorbidity is common, with associated depression, anxiety, post-traumatic stress disorder, self-harm, and suicidality frequently reported in this population (12, 13). Given this burden of psychiatric illness, suicide risk should be treated as a priority concern in all FDS assessments, particularly when depression, trauma history, and self-harm are present together.

### 2.3 Trauma, attachment, and dissociation

Early adversity, sexual trauma, and disrupted attachment are among the most consistently reported vulnerability factors for FDS (14–16). Attachment theory frames such adversity as a disruption of the relational systems that normally support affect regulation, rendering individuals more vulnerable to emotional dysregulation when subsequent relationships reactivate early experiences of fear, helplessness, shame, or abandonment (17). Dissociation provides a mechanistic link between trauma and seizure generation: when emotional distress exceeds tolerable limits, dissociative processes may disrupt conscious awareness and translate psychological distress into physical symptoms (18–20). Shame and trauma-related perceptual intrusions are common and may, at the threshold of an episode, be misread as primary psychosis.

### 2.4 Cultural explanatory models in Ethiopia

Help-seeking and meaning-making for seizure-like episodes in Ethiopia are shaped by culturally available explanatory models, including Zar and azurit possession, Buda and the evil eye, and religious healing through holy water, prayer, and exorcism (5, 21, 22). Across sub-Saharan Africa, religious and traditional pathways frequently precede or substitute for biomedical care for epilepsy and functional disorders (6, 23). Such frameworks can reduce self-blame and confer meaning, but exclusive reliance on them may delay assessment, reinforce fear, and expose vulnerable patients to social or financial harm. A culturally responsive approach acknowledges and respects the patient’s explanatory model while gradually introducing a neuropsychiatric understanding.

### 2.5 Structural violence, gender, and diagnostic infrastructure

FDS in this population cannot be separated from the broader structural conditions in which it occurs. Structural violence, defined as the systematic disadvantage embedded in poverty, gender, and institutional neglect, shapes both vulnerability to trauma and access to care (24). In Sub-Saharan Africa, including rural Ethiopia, the diagnosis and management of seizure disorders is constrained by limited access to EEG, specialist neurology, and adequate diagnostic infrastructure, with primary health care workers in Ethiopia demonstrating low detection of comorbid mental disorders in people with epilepsy and lacking the resources and training required to identify non-epileptic seizures (25, 26). To quantify this scarcity, Ethiopia has approximately one neurologist per 1.6 million people, most of whom practice in Addis Ababa (27); routine EEG is limited and largely confined to the capital, while video-EEG and other advanced electrophysiological modalities are rarely available. The result is systemic invisibility: a treatable functional neurological condition that is rarely named, rarely measured, and rarely followed up. Addressing this gap requires culturally adapted assessment pathways, integrated trauma-informed services, and dedicated Ethiopian FDS research priorities to which this case series is intended to contribute.

### 2.6 Medical comorbidity and biomedical vulnerability

FDS does not occur in a purely psychological vacuum; physical health and biomedical factors can lower the threshold for functional seizure-like episodes and must be considered both in the explanatory model and at the bedside. General medical conditions and physiological states—including sleep deprivation, intercurrent infection or febrile illness, anaemia and nutritional deficiency, pain, head injury, the physiological effects of substances such as khat and alcohol and their withdrawal, and the adverse effects of prescribed medication—can destabilize arousal regulation and interact with trauma-related vulnerability. Comorbid neurological disease, including epilepsy, may also coexist with FDS rather than excluding it. Accordingly, in this series each woman underwent general physical and neurological examination together with review of any available investigations, and identified medical contributors were treated or referred as part of routine care (Section 3.3); the residual diagnostic uncertainty arising from the absence of electrophysiological testing is addressed in Sections 3.4 and 7.

## 3 Setting and methods

### 3.1 Setting

The cases were identified among residents of the Lenegewa Women’s Rehabilitation and Skills Development Center in Addis Ababa during routine clinical screening and individual consultations between October 2024 and April 2026. The Center provides a residential rehabilitation and vocational-training program for young women, many with histories of trauma exposure, social marginalization, economic vulnerability, and disrupted family support. The six women in this series were aged 18–24 years (median 20). None had received a prior diagnosis of FDS. In the Ethiopian clinical context, FDS is not routinely recognized as a diagnostic category, and seizure-like episodes are commonly attributed to epilepsy, spirit possession, evil eye, Zar, Azurit, or acute psychiatric illness.

### 3.2 Case identification and eligibility

Eligible cases met the following inclusion criteria: recurrent episodes of altered consciousness, altered responsiveness, or motor activity described by the patient or witnesses as seizure-like; no prior diagnosis of FDS; engagement in clinical assessment and treatment at the Center; and written informed consent for the use of de-identified data. Exclusion criteria were confirmed epilepsy by prior EEG, acute psychotic disorder, or inability to provide informed consent. All identifiers were abbreviated or removed before analysis. This is a retrospective analysis of clinical records generated during routine care.

Although all 3,617 women in the Center’s client population underwent systematic, staff-supported behavioral-health screening at intake—including the PHQ-9, GAD-7, an adapted ACE measure, the CAGE, and the SDS—the present cases were not ascertained through that screening. The women in this series were among the approximately 1 to 1.5% who presented to the medical team with recurrent episodes described by themselves or by witnesses as seizure-like and who were then clinically assessed for inclusion. No record was kept of the number subsequently excluded for confirmed epilepsy on prior EEG or for acute psychosis. The six cases reported here are among the approximately 25 to 30 women referred for mental health services by the medical team during the period.

Because identification occurred within a large, systematically screened cohort in which functional/dissociative seizures were not recognized as a diagnostic category, and in which seizure-like episodes were routinely attributed to epilepsy, spirit possession, the evil eye, Zar, azurit, or acute psychiatric illness, the observation that these presentations are under-recognized is grounded in the screening context rather than asserted from the six cases alone. At the same time, ascertainment relied on clinical presentation and behavioral-health referral rather than neurological or video-EEG evaluation, so the count of six reflects clinically recognized cases within one service during the period and is not an epidemiological estimate of the true frequency of functional/dissociative seizures in the underlying population. We therefore treat the series as illustrative of a clinical pattern that is plausibly relevant to comparable high-adversity female rehabilitation populations, while avoiding any claim of national or general-population generalizability; this sampling frame and its bearing on inferences about under-recognition and transferability are revisited in the Limitations (Section 7).

### 3.3 Clinical assessment and measures

Clinical assessment included a psychiatric interview, trauma history, mental-status examination, episode history, witness descriptions where available, and review of available clinical information. Standardized measures comprised the Adverse Childhood Experiences (ACE) questionnaire adapted for the Ethiopian context; the Patient Health Questionnaire-9 (PHQ-9); the Generalized Anxiety Disorder-7 (GAD-7); the CAGE questionnaire for alcohol-related risk; and the Severity of Dependence Scale for khat (SDS-Khat) and other locally relevant substances. Substance-use screening was interpreted within the broader formulation and was not used as a stand-alone explanation for seizure-like episodes.

Measures were administered in Amharic by clinical staff fluent in the language. The PHQ-9 and GAD-7 have been translated into Amharic and applied in Ethiopian populations; the ACE questionnaire was administered in an interviewer-assisted format adapted to the local context rather than as an English-language self-report; and the CAGE and SDS-Khat items were completed through clinician interview. Scores were interpreted against established cut-points: for the PHQ-9, thresholds of 5, 10, 15, and 20 demarcate mild, moderate, moderately severe, and severe depression; for the GAD-7, thresholds of 5, 10, and 15 demarcate mild, moderate, and severe anxiety; for the CAGE, a score of two or more indicates clinically significant alcohol-related risk; and for the ACE questionnaire, a score of four or more denotes high cumulative childhood adversity. For readability and to limit re-identification risk in this small all-female sample, screening results are presented in the case descriptions as the corresponding severity band rather than as raw totals; the exact item-level and total scores were recorded in the clinical file and can be provided on reasonable request.

For every included case, at least one seizure-like episode was witnessed directly by trained residential clinical staff during the period of care, working under the supervision of the attending clinician; for events that occurred before admission or outside staffed hours, additional descriptions were obtained from the patient and from family members or friends who had witnessed them. The qualifier “where available” therefore refers to these pre-admission or out-of-facility events, and the per-case source of episode observation is indicated in the case presentations. In addition, each woman underwent general physical and neurological examination, with review of any available laboratory and clinical investigations, to identify medical or neurological contributors and to inform the differential diagnosis; the examination findings relevant to each case are summarized in the formulations and in Section 3.4.

Episodes typically presented as a semi-emergency: an epilepsy-like event on the residential campus prompted a medical officer—a nurse or a physician—to be called to the scene, or a group of residents brought the affected woman to the on-site medical center. These first responders provided acute medical assessment and stabilization; they were general nursing and medical staff without specific training in functional or dissociative seizures. Once the woman was medically stable, she was referred for emergency mental-health assessment to the Center’s mental-health team, composed of physicians with extensive training in trauma-informed care and supervised by a psychiatrist. This team held no formal qualification specific to functional neurological disorder; its competence in recognizing and managing FDS was developed through structured literature review and ongoing clinical supervision, an adaptation the setting required as such presentations recurred.

### 3.4 Diagnostic approach

**Figure 2:**
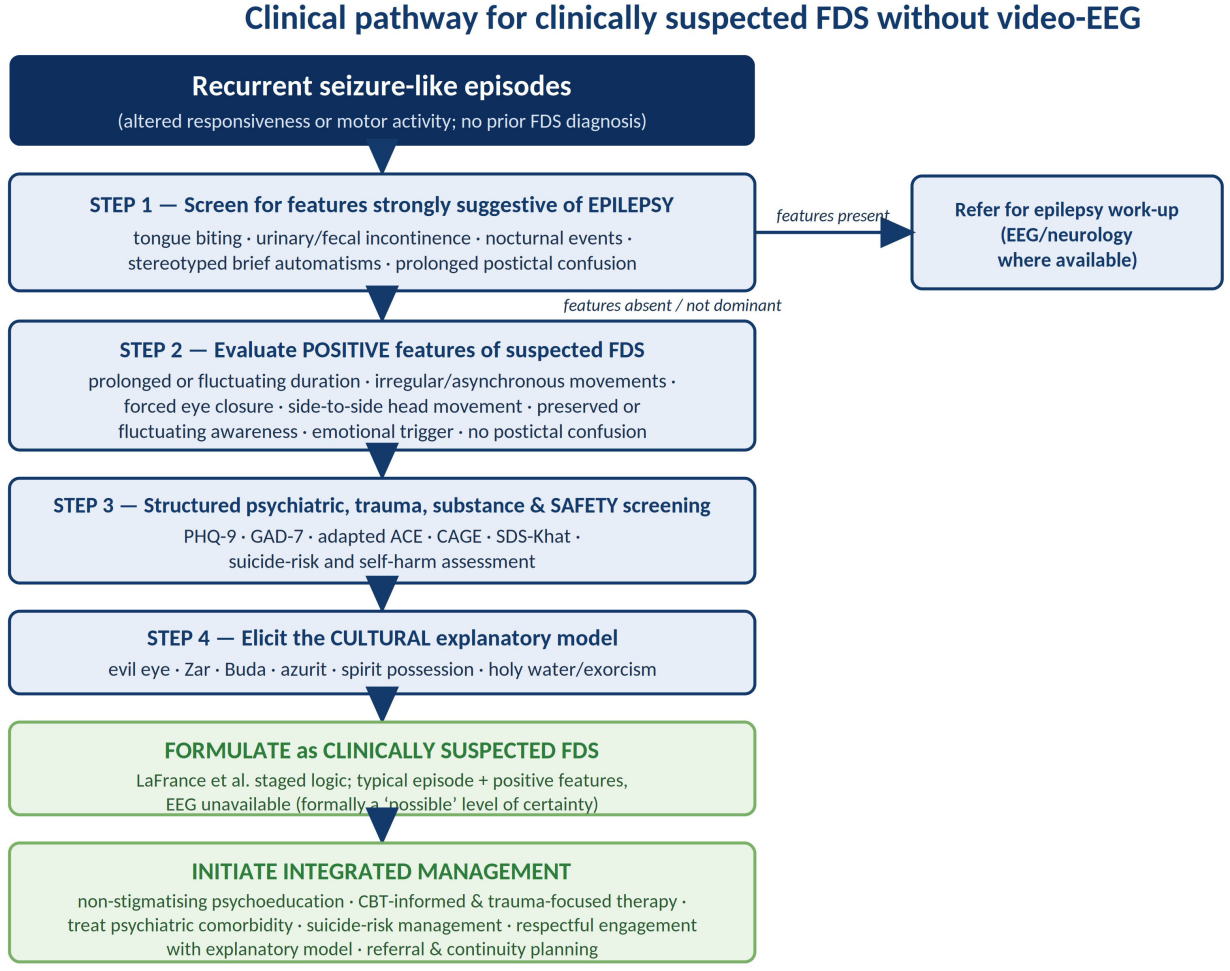
Clinical pathway for identifying clinically suspected FDS where video-electroencephalography is unavailable. Original figure created by the authors for this study, informed by the staged diagnostic criteria described in LaFrance et al. 2013 (28).

Because no EEG (routine, ambulatory, or video) was available at the Center or through the referral pathway during the assessment period, diagnosis followed the staged logic of LaFrance et al. (28) adapted to a resource-limited setting (**Figure 2**). Episodes were evaluated for positive clinical features suggestive of FDS, prolonged or fluctuating duration, irregular or asynchronous motor activity, forced eye closure, side-to-side head movement, preserved or fluctuating awareness, absence of postictal confusion, and emotional or interpersonal triggers and for features strongly suggestive of epilepsy, such as stereotyped brief automatisms, tongue biting, urinary or fecal incontinence, nocturnal episodes, or prolonged postictal confusion.

Beyond episode semiology, the differential diagnosis was approached actively rather than by inference from the absence of epileptic features alone. Each woman underwent general physical and neurological examination, and available investigations were reviewed to evaluate alternative or contributing causes of transient loss of consciousness or altered responsiveness, including acute intoxication or withdrawal (alcohol, khat, and other substances), metabolic and systemic disturbances (such as hypoglycaemia, electrolyte imbalance, anaemia, and infection), syncope, and adverse medication effects. Current medications were reviewed for agents that lower the seizure threshold or alter arousal. Where the history, examination, or clinical course raised concern for epilepsy or for a primary medical or structural cause, neurological referral and electrophysiological evaluation were sought; as detailed in Sections 3.1, 2.5, and 7, such referral and EEG were not accessible within the assessment period because of cost, distance, and the national shortage of neurological services. The diagnosis offered here is therefore explicitly provisional: we do not claim that epilepsy or a comorbid medical cause was definitively excluded, and the cases are accordingly described as clinically suspected rather than confirmed.

We are explicit about diagnostic certainty. Under the formal LaFrance scheme, the four ascending levels (possible, probable, clinically established, documented) each require progressively stronger electrophysiological and witnessed-event evidence. Because no EEG data were available, these cases cannot be formally classified beyond the *possible* level. We use the descriptive term *clinically suspected FDS* throughout to denote cases in which positive FDS features clearly predominate, epilepsy features are absent, and trained residential clinical staff directly observed typical episodes, a level of clinical confidence that strengthens, but does not substitute for, electrophysiological confirmation. Epilepsy and mixed seizure disorders cannot be fully excluded.

### 3.5 Analytic approach

The case series was analyzed using thematic analysis following Braun and Clarke (29), organized around pre-established conceptual domains from Section 2 in a codebook/framework approach rather than fully inductive reflexive analysis. Each de-identified case narrative served as the unit of analysis, encompassing episode features, psychiatric and neuropsychiatric presentation, trauma and attachment history, socioeconomic and gendered context, cultural explanatory models, treatment pathway, and short-term outcome. Coding proceeded in stages: familiarization with each narrative; application of an initial codebook derived from the Section 2 domains (**Table 1**); inductive addition of codes for patterns not anticipated by the framework; collation of codes into candidate themes; and review of themes against the full dataset. Two clinicians independently coded each narrative; discrepancies were resolved through discussion and, where needed, adjudicated by a third senior author. The analysis is interpretive and clinically grounded rather than statistical; the reported theme prevalence reflects only this small purposive sample and is not an epidemiological estimate.

**Table 1:** Analytic domains, case-level data elements, and their interpretive purpose.

Analytic domain	Case-level data elements reviewed	Interpretive purpose
Episode semiology	Duration; motor pattern; awareness; eye closure; postictal state; injury; nocturnal occurrence; triggers	Differentiate suspected FDS features from epileptic features where EEG was unavailable (Section 2.1, 3.4)
Psychiatric/neuropsychiatric presentation	Mood; anxiety; suicidality; self-harm; dissociation; perceptual phenomena; personality features; cognition	Clarify comorbidity, risk, and mechanism (Section 2.2, 2.3)
Trauma and attachment history	Abandonment; sexual abuse; forced marriage; homelessness; separation from child; family rejection	Link individual vulnerability to the trauma–attachment–dissociation pathway (Section 2.3)
Socioeconomic and gendered context	Poverty; lack of family protection; dependency; limited education; social shame; structural marginalization	Identify structural violence and gendered vulnerability as maintaining conditions (Section 2.5)
Cultural and religious attribution	Zar; Azurit; Buda; evil eye; spirit possession; holy water; exorcism; prayer pathways	Analyze meaning-making, help-seeking, treatment delay, and potential harm (Section 2.4)
Treatment and outcome	Psychoeducation; CBT; trauma-focused work; medication; suicide monitoring; discharge referral	Evaluate short-term response and continuity-of-care limitations (Section 2.2, 2.5)

The codebook combined a deductive framework with inductive elaboration. The deductive scaffold was specified *a priori* from the conceptual domains set out in Section 2 and operationalised as ten code families: neurological episode features; neuroscience mechanism; affective trigger; dissociation; psychiatric risk; trauma and attachment; substance-related factors; cultural attribution; structural context; and treatment and outcome (**Table 2**). Each family was given a working definition with inclusion and exclusion notes and at least one anchoring example, so that both coders applied it consistently; for example, the neurological-episode family captured directly observed or reported seizure-semiology features (prolonged or irregular movements, side-to-side head movement, forced eye closure, fluctuating awareness, sensory prodrome, rapid recovery, and the absence of tongue-biting, incontinence, or nocturnal events), whereas the dissociation family captured amnesia, limited recall, peri-episodic perceptual phenomena, and inability to respond despite partial awareness. The unit of coding was the meaning unit (a phrase, sentence, or short passage conveying a single idea) within each de-identified case narrative, and a single unit could receive codes from more than one family where its content overlapped. The codebook was maintained as a shared, version-dated document in which every code carried its definition and at least one illustrative case-level instance; **Table 2** reproduces the final version, pairing each code family with representative case-level codes and the focused analytic category to which it contributed.

**Table 2:** Coding framework: code families, illustrative case-level codes, and focused analytic categories.

Code family	Illustrative case-level codes	Focused analytic category
Neurological episode	Prolonged/irregular/asynchronous movements; side-to-side head movement; forced eye closure; fluctuating awareness; sensory prodrome; rapid recovery; no postictal confusion; no nocturnal/tongue-biting/incontinence	Suspected functional/dissociative seizure pattern
Neuroscience mechanism	Threat arousal; interoceptive warning; impaired top-down regulation; limbic activation; motor expression of distress; functional shutdown	Limbic-motor and interoceptive dysregulation
Affective trigger	Rejection; peer conflict; romantic loss; grief; nightmares; perceived abandonment; shame; family conflict	Affective overload and attachment activation
Dissociation	Amnesia; limited recall; peri-episodic perceptual phenomena; emotional exhaustion; inability to respond despite partial awareness	Trauma-related dissociation and shutdown
Psychiatric risk	Suicide attempt; self-harm; hopelessness; worthlessness; depression; anxiety; nightmares; emotional lability; personality features	Psychiatric comorbidity and safety risk
Trauma and attachment	Parental abandonment; sexual abuse; child marriage; homelessness; maternal rejection; estrangement; separation from child	Developmental adversity and attachment rupture
Substance-related	Prior alcohol/khat/cannabis use; intermittent khat; sobriety at assessment; sleep and arousal effects	Substance use as vulnerability modifier
Cultural attribution	Evil eye; Buda; Zar; azurit; spirit possession; holy water; church rituals; exorcism	Cultural meaning-making and treatment pathway
Structural context	Poverty; unstable housing; gendered control; social shame; no EEG; no prior diagnosis; fragmented referral	Structural violence and systemic invisibility
Treatment and outcome	Psychoeducation; CBT-informed therapy; trauma-focused work; supportive therapy; fluoxetine; suicide watch; referral; episode reduction; relapse risk	Multidisciplinary response and continuity needs

**Figure 3:**
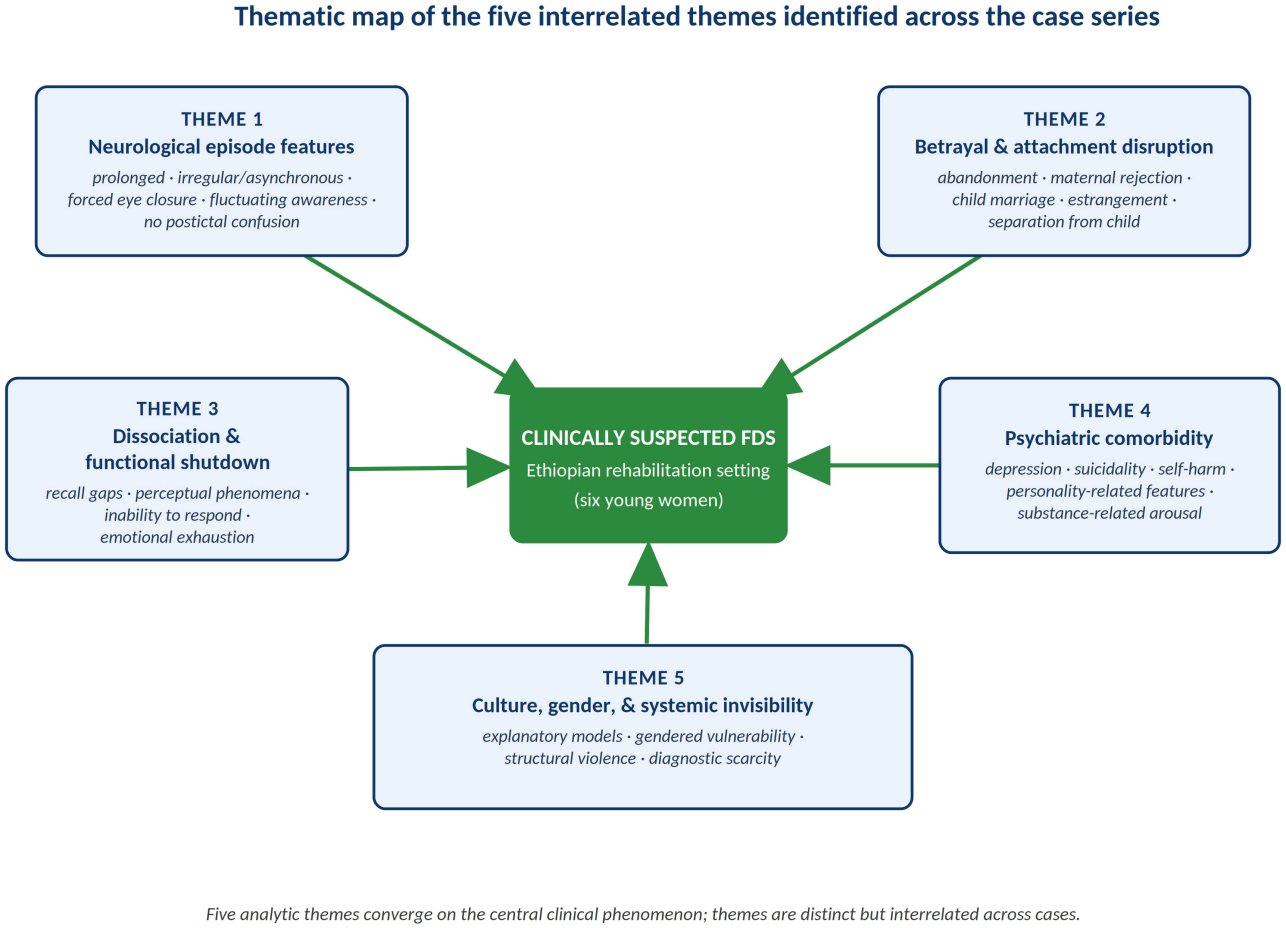
Thematic map of the five interrelated themes identified across the case series. Solid spokes link each theme to the central phenomenon; dashed lines indicate the clinical interdependence of themes within individual cases.

Coding then proceeded in defined, auditable steps. After familiarisation through repeated reading of all six narratives, two clinicians independently coded each narrative against the *a priori* codebook. Coding was iterative and comparative: when a meaning unit did not fit any existing code, the coder proposed a candidate inductive code with a working definition, which the team then reviewed, defined formally, and—if retained—added to the codebook, after which previously coded narratives were re-examined against the new code (constant comparison) so that codes were applied uniformly across the series. The inductively added codes were those not anticipated by the Section 2 framework, including peri-episodic perceptual phenomena (voice-like experiences bound to the threshold of an episode), the holy-water and monastery treatment pathway, sensory prodrome, and documented sobriety at the time of assessment. Independent coding produced two parallel coding sets for each narrative; these were compared, agreements were retained, and every disagreement was logged and resolved in a reconciliation meeting—first by discussion to consensus and, where consensus could not be reached, by adjudication from a third senior author—with the basis for each final decision recorded and, where it clarified a definition, written back into the codebook. Coded units were then collated into candidate themes, reviewed against the full dataset for internal coherence and external distinctiveness, and defined and named; the five resulting themes are mapped in **Figure 3** and their cross-case distribution is summarised in **Table 3**. Throughout, analytic decisions were supported by reflexive memos and interpreted in light of the team’s stated positionality (Section 3.6), and reporting follows the Standards for Reporting Qualitative Research (30).

**Table 3:** Cross-case theme matrix: evidence, neuropsychiatric interpretation, and cultural/systemic interpretation.

Theme	Cases	Core clinical evidence	Neuropsychiatric interpretation	Cultural/systemic interpretation
Neurological episode features	All six	Prolonged events; irregular/asynchronous movements; forced eye closure; sensory prodrome; recall gaps; no postictal confusion	Involuntary disruption of awareness, motor control, interoception, threat processing (Section 2.1)	Absence of EEG and limited recognition drive diagnostic uncertainty
Betrayal and attachment disruption	All six	Abandonment; maternal rejection; child marriage; restricted childhood; estrangement; separation from child	Attachment rupture increases dysregulation, rejection sensitivity, and dissociation vulnerability (Section 2.3)	Gendered dependency, poverty, and family rejection intensify vulnerability
Dissociation and functional shutdown	All six	Recall gaps; emotional overwhelm; peri-episodic perceptual phenomena; emotional exhaustion	Dissociative/functional shutdown when arousal exceeds regulatory capacity (Section 2.3)	Dissociation may be misinterpreted as psychosis, Zar, azurit, or the evil eye
Psychiatric risk and comorbidity	All six; highest in M.S., R.H.	Depression; suicidality; self-harm; nightmares; anxiety; personality features; khat use	FDS within broader psychiatric syndromes; suicide risk and comorbidity require active treatment (Sections 2.2, 2.3)	Limited psychiatric access leaves mood, trauma, and safety untreated
Culture, gender, and systemic invisibility	All six	Evil eye; Zar; azurit; spirit possession; holy water; no prior diagnosis; no EEG	Cultural models shape meaning but do not preclude a neuropsychiatric formulation (Section 2.4)	Spiritual pathways may reduce shame but can delay care; gendered structural violence sustains invisibility (Section 2.5)

Three distinct levels of evidence are combined in the analysis and are distinguished here to avoid conflating them. First, established findings from the published literature, summarized in the Conceptual Framework (Section 2)—including predictive-processing and limbic models of functional seizures, trauma–attachment–dissociation pathways, and the concept of structural violence—are claims drawn from prior research, not findings of the present study. Second, directly recorded clinical data—episode descriptions, mental-status findings, trauma histories, screening severity bands, treatments given, and observed short-term course—are the primary observations of this case series. Third, the neuropsychiatric and sociocultural formulations that connect the two—for example, interpreting a given woman’s episodes as limbic-motor dysregulation under attachment threat, or reading a cultural attribution as shaping help-seeking—are the authors’ interpretive synthesis. Mechanistic constructs such as predictive-processing failure, limbic-motor activation, and structural violence are therefore used as explanatory lenses applied to clinical observation, not as variables that were directly measured in these patients.

### 3.6 Reflexivity and positionality

Consistent with reporting standards for qualitative research (30), we state our positionality. The analytic team comprised Ethiopian clinicians working in rehabilitation settings and diaspora-based clinical and academic collaborators with backgrounds in psychiatry, clinical psychology, and global mental health. Several authors deliver the care described, which affords contextual and linguistic access to patients’ explanatory models but also carries a risk of confirmation bias toward a neuropsychiatric reading of the episodes. We mitigated this through independent dual coding, explicit attention to disconfirming features (including features that might indicate epilepsy), and retention of patients’ own cultural explanations as primary data rather than as artifacts to be corrected. Our shared commitment to making functional neurological disorder visible in Ethiopia is a motivating standpoint that we make explicit so that readers can weigh it when interpreting our claims.

## 4 Case presentations with multidisciplinary neuropsychiatric formulation

Six cases are presented. Each follows a common structure — presentation and episode pattern; background and trauma history; mental-status examination; standardized screening; culturally informed formulation; treatment; and outcome — with formulations referring to the framework in Section 2. **Table 4** summarizes the six cases' episode features, neuropsychiatric profiles, and cultural attributions.

**Table 4:** Cross-case clinical summary: episode features, neuropsychiatric profile, and cultural attribution.

Case (age)	Key episode features	Neuropsychiatric profile and screening	Context and cultural attribution
E.A. (19)	Burning ear/tinnitus prodrome; altered responsiveness; arm/leg shaking; no postictal confusion	Mild depression/anxiety; high ACE; restricted affect; partial dissociative recall gaps; peer-conflict trigger	Parental abandonment; economic dependency; evil-eye attribution; no EEG; no prior diagnosis
B.A. (18)	Abrupt collapse; ~10-min awareness gap; minimal motor activity; emotional discharge on recovery	Moderate depression/anxiety; sexual abuse; homelessness; prior polysubstance use; self-harm history; rejection sensitivity	Extreme poverty; no adult protection; gendered street vulnerability; no prior FDS pathway
M.S. (23)	Unresponsiveness with eye closure; limited recall; peri-episodic voices; no postictal confusion	Moderate depression with suicide risk; two prior attempts; relational loss; dissociative (not psychotic) intrusions	Spirit-possession attribution; holy-water/monastery pathway; unstable housing; minimal support
B.Z. (21)	Prolonged variable shaking; immediate recovery; dissociative recall gaps; no postictal confusion	Moderate depression; high ACE; child sexual abuse within child marriage; emotional numbing; attention difficulty	Child marriage at 12; family estrangement; rural poverty; spirit-possession attribution
B.S. (24)	Irregular/asynchronous movements; side-to-side head movement; forced eye closure; fluctuating awareness; rapid recovery	Low PHQ-9/GAD-7; provisional histrionic features (held cautiously); relational dysregulation; restricted social development	Gendered childhood control; limited autonomy; evil-spirit attribution; holy-water rituals
R.H. (19)	Sudden unresponsiveness; irregular movements; prolonged episodes; limited recall; emotional exhaustion	Moderate depression with suicide risk; sexual abuse; nightmares; unresolved grief; intermittent khat use	Separation from child; family dependency; social shame; Zar/azurit attribution; no clear prior diagnosis

No EEG of any kind was available for any case; under the LaFrance et al. (28) scheme the formal level is “possible,” and “clinically suspected” is used descriptively (see Section 3.4). Trained residential staff directly observed typical episodes in all cases.

### 4.1 Case 1: E.A. | 19 years | female

#### 4.1.1 Presentation and episode pattern

E.A. was brought by friends after five months of recurrent episodes of loss of consciousness, during which she spoke incoherently and shouted with no subsequent memory. On one occasion, she climbed onto an elevated ledge, raising concern for possible self-harm intent. Episodes began with a warning phase of burning ear pain and tinnitus, followed by visible shaking of the arms and legs witnessed by friends; she then became unresponsive, although her eyes remained open. Afterward, she was fatigued, irritable, and tearful. Episodes resolved spontaneously, lasted 8–20 minutes, and occurred two to three times per week since admission. No EEG was available. E.A. attributed her episodes to a specific peer acting through the “evil eye.” She also reported mild, sub-threshold anxiety and depressive symptoms.

#### 4.1.2 Background and trauma history

E.A.’s parents separated when she was seven, after which she lost contact with both for approximately seven years; she was raised by her paternal grandmother in a crowded government shelter. Contact with her mother was later re-established, while she remained estranged from her father, whom she experienced as absent and unloving. Her grandmother, a gardener, was her primary caregiver and sole financial support. She completed Grade 12 but did not qualify for university, which she described as a significant disappointment.

#### 4.1.3 Mental-status examination

E.A. was appropriately dressed and groomed, calm but guarded. Her self-described mood was “sad” with a congruent dysphoric affect. Speech was normal; thought process was goal-directed; she was fully oriented with intact attention and memory. No perceptual abnormalities were reported. Insight was Level 3 — awareness of being unwell while attributing symptoms to an external individual.

#### 4.1.4 Standardize screening

PHQ-9: Mild.

GAD-7: Mild.

ACE score: High.

CAGE: Low risk.

SDS-Khat: Low risk.

#### 4.1.5 Examination and investigations

General physical and neurological examination did not reveal focal neurological deficits or signs of an acute medical illness. No blood, urine, or cerebrospinal-fluid testing, neuroimaging, or electroencephalography was performed; the assessment rested on history, directly observed episode semiology, and clinical examination, consistent with the resource constraints set out in Section 3.4. No antidepressant, antipsychotic, antiepileptic, or other medication known to alter the seizure threshold was prescribed.

#### 4.1.6 Culturally informed neuropsychiatric formulation

E.A.’s clinical presentation is consistent with clinically suspected functional/dissociative seizures (FDS) within the broader framework of Functional Neurological Disorder. The episodes were recurrent, prolonged, stress-associated, and characterized by altered responsiveness, spontaneous resolution, and absence of postictal confusion. Although formal video-EEG confirmation was not available, the observed clinical episode features are more consistent with functional or dissociative seizure-like episodes than with epileptic seizures. Her presentation should therefore be understood as clinically suspected FDS rather than a confirmed diagnosis.

Several observed features of the episodes support this formulation. E.A. reported a consistent warning phase involving burning ear pain and tinnitus before becoming unresponsive. These prodromal sensory symptoms may occur when internal bodily sensations become linked to the anticipation of threat. The episodes lasted several minutes to approximately twenty minutes, resolved spontaneously, and were followed by fatigue but not the sustained postictal confusion typically expected after generalized tonic-clonic seizures. There was also no tongue biting, urinary or fecal incontinence, nocturnal occurrence, or injury pattern strongly suggestive of epilepsy. The frequency of episodes, their relationship to interpersonal stress, and the absence of a clear epileptic recovery pattern support a functional neurological formulation.

E.A.’s developmental history provides an important vulnerability pathway. Her vulnerability did not arise from a single event but from the cumulative weight of adversity across childhood, including parental abandonment at age seven, upbringing in a crowded extended household with limited stability, chronic insecurity, and restricted access to consistent emotional support. As discussed in Section 2.3, early adversity and attachment disruption are strongly associated with vulnerability to FDS, particularly when later stressors activate unresolved relational fear, helplessness, or abandonment-related distress. In E.A.’s case, peer conflict within the residential environment appears to have functioned as a key precipitating and maintaining factor. Her belief that a peer caused her illness through the “evil eye” suggests that interpersonal threat was not experienced as ordinary conflict, but as a serious danger to bodily and psychological safety.

From the neurobiological perspective outlined in Section 2.1, E.A.’s episodes can be understood as involuntary functional responses that arise when interpersonal stress activates threat-processing systems beyond her capacity for voluntary regulation. Early abandonment and chronic insecurity may have sensitized limbic threat circuits, increasing vulnerability to heightened bodily arousal and altered motor control under stress. In this framework, the prodromal burning ear pain and tinnitus may represent interoceptive warning signals associated with impending emotional overload. When threat arousal becomes overwhelming, the brain may generate involuntary seizure-like changes in motor activity and awareness through altered limbic-motor and interoceptive processing, rather than through epileptiform electrical activity. From the dissociation and psychiatric-comorbidity framework discussed in Section 2.3, E.A.’s partial memory gaps and difficulty recalling episode details are best understood as dissociative phenomena rather than evidence of organic memory impairment.

Her attribution of the episodes to the evil eye should be interpreted within the cultural explanatory models described in Section 2.4. This belief does not indicate delusional thinking. Rather, it reflects a locally meaningful framework for explaining sudden collapse, altered consciousness, and seizure-like behavior. At the same time, when evil-eye attribution becomes the primary or exclusive explanation, it may reinforce fear of peers, intensify hypervigilance, and delay engagement with psychological or neurological care. A clinically appropriate approach should therefore respect the cultural meaning of her explanation while gradually introducing a neuropsychiatric formulation that links interpersonal stress, bodily arousal, dissociation, and functional seizure-like episodes.

E.A.’s socioeconomic context also contributes to the formulation. Her grandmother’s household survived through daily labor, and E.A. had no independent income. As a young woman without higher education, stable family support, or economic security, she faced limited options for autonomy and social mobility. These conditions likely intensified her dependence on the residential environment and reduced her ability to escape triggering interpersonal dynamics. The ongoing presence of peers whom she believed had caused her illness may therefore have functioned as a maintaining stressor, increasing anticipatory anxiety and reinforcing the episode cycle.

Finally, E.A.’s lack of access to EEG evaluation and absence of a prior FDS diagnosis reflect the diagnostic infrastructure limitations described in Section 2.5. Her case illustrates how suspected FDS may remain invisible in Ethiopia when seizure-like episodes are interpreted primarily through epilepsy or spiritual frameworks and when video-EEG, specialized neurology, and trauma-informed psychiatric assessment are unavailable. Consistent with the research priorities outlined in Section 2.5, her presentation underscores the need for culturally adapted FDS assessment pathways that can integrate episode features, trauma history, psychiatric symptoms, cultural attribution, and socioeconomic vulnerability. E.A. also had meaningful protective factors. Her grandmother provided the most consistent attachment relationship in her history, and her religious faith offered a framework for meaning-making and emotional coping.

#### 4.1.7 Treatment

Treatment combined psychoeducation about the functional nature of the episodes, sleep hygiene, and grounding and breathing strategies with weekly individual therapy. Cognitive Behavioral Therapy (CBT) addressed the links between emotional distress, interpersonal stressors, and episodes, with attention to abandonment and rejection; trauma-focused elements addressed early parental separation. Interpersonal techniques targeted peer conflict, communication, and conflict resolution. Given mild comorbidity and identifiable psychosocial stressors, medication was not initiated.

#### 4.1.8 Outcome

Episode frequency fell progressively; by her final month she was episode-free, compared with two to three per week at admission. She developed early awareness of triggers and the link between emotional stress and physical symptoms. She was referred to outpatient mental-health services on graduation. Given her trauma history and the absence of structured post-exit follow-up, relapse risk remains high.

### 4.2 Case 2: B.A. | 18 years | female

#### 4.2.1 Presentation and episode pattern

B.A. presented to the emergency department, accompanied by friends, after one year of recurrent sudden loss of consciousness, initially two to three times per month and rising to once or twice weekly over five months — the escalation coinciding with a phone call in which her mother told her never to call again. She described dizziness, headache, and overwhelming feelings of rejection and anger before each episode, which typically followed feeling misunderstood or rejected by peers or staff. Loss of consciousness lasted about ten minutes; observers noted an abrupt collapse with trembling of both arms, no stiffness, no injury, no incontinence, and no tongue biting. On recovery, she screamed, cried, and became agitated before settling.

#### 4.2.2 Background and trauma history

B.A.’s parents divorced at her birth; her father’s whereabouts are unknown. Her mother left Ethiopia for work abroad, and B.A. was raised by a maternal aunt. Contact with her mother was inconsistent. She began using substances in high school — cigarettes and alcohol, later khat. After her aunt discovered this and informed her mother, conflict led B.A. to leave home, beginning roughly three years of street homelessness in Addis Ababa. She had a history of non-suicidal self-harm by cutting. On the streets, her substance use escalated to glue and tramadol; an attempt to seek financial help from her mother was refused. Community members eventually brought her to a Missionaries of Charity center, where she received about three months of care; she had been sober for three months at assessment, and during this period disclosed sexual abuse at age 13.

#### 4.2.3 Mental-status examination

B.A. was appropriately dressed and anxious, with good eye contact but initially guarded and withdrawn behavior. Speech was normal. Mood was sad; affect was dysphoric and labile. Thought content showed hopelessness, worthlessness, and guilt, with no suicidal ideation elicited. There were no perceptual disturbances. She was fully oriented with intact attention; memory was intact except for episode content. Insight was Level 4 — she accepted a psychological contribution but had limited capacity to apply this awareness in the moment.

#### 4.2.4 Standardize screening

PHQ-9: Moderate depression.

GAD-7: Moderate anxiety.

ACE score: High (parental absence from birth, homelessness, sexual abuse at 13, parental rejection, prolonged polysubstance use).

CAGE: Moderate risk.

SDS-Khat: Mild dependence.

#### 4.2.5 Examination and investigations

General physical and neurological examination did not reveal focal neurological deficits or signs of an acute medical illness. No blood, urine, or cerebrospinal-fluid testing, neuroimaging, or electroencephalography was performed; the assessment rested on history, directly observed episode semiology, and clinical examination, consistent with the resource constraints set out in Section 3.4. The seizure-like episodes preceded any pharmacotherapy; fluoxetine, a selective serotonin reuptake inhibitor, was started only afterwards as treatment for depression, and no antipsychotic or antiepileptic medication was used.

#### 4.2.6 Culturally informed neuropsychiatric formulation

B.A.’s presentation is consistent with clinically suspected functional/dissociative seizures (FDS) within the broader framework of Functional Neurological Disorder, occurring against a background of severe developmental trauma, disrupted attachment, emotional dysregulation, and heightened sensitivity to relational threat. At admission, episodes occurred multiple times per week and were consistently triggered by situations in which she felt misunderstood, rejected, criticized, or emotionally abandoned by peers or staff. This pattern suggests that the episodes were involuntary functional responses to interpersonal stress that exceeded her regulatory capacity, rather than random neurological events or intentional behavior.

B.A. described rapid emotional overwhelm immediately before each episode, followed by a gap in consciousness lasting approximately ten minutes. Observers noted minimal motor activity rather than a stereotyped tonic-clonic pattern, and recovery was marked by intense emotional release (screaming and crying) rather than postictal confusion. Although EEG evaluation was unavailable at the referral center, the overall pattern (clear emotional triggers, altered awareness, limited recall, minimal motor activity, affective discharge on recovery) was less typical of epileptic seizures and more consistent with functional or dissociative seizure-like episodes. From the neurobiological perspective outlined in Section 2.1, relational threat activates the limbic system and overwhelms top-down regulatory control, causing emotional arousal to bypass reflective regulation and manifest as a dissociative shutdown or seizure-like events.

B.A.’s developmental history represents one of the most severe profiles of adversity in this case series. She experienced sexual abuse at age 13, prolonged abandonment, maternal rejection, street homelessness, substance exposure, and the sustained absence of adult protection. As discussed in Section 2.3, early adversity, sexual trauma, abandonment, and disrupted attachment are strongly associated with FDS vulnerability, particularly when later interpersonal stress reactivates unresolved fear, shame, helplessness, and rejection sensitivity. In B.A.’s case, perceived rejection appears to function as a central trigger not merely as disappointment but as reactivation of earlier abandonment and relational injury.

B.A.’s history of alcohol, khat, cannabis, and other substance use during street homelessness should be understood within the context of survival, trauma exposure, and lack of protection. Substance use may have functioned as an attempt to regulate distress, numb traumatic memories, or cope with street vulnerability. Although she was 3 months sober at the time of assessment, her substance-use history remains clinically relevant because it reflects the severity of her prior social exposure and the need for continued relapse-prevention support. It also increases the importance of integrated treatment addressing trauma, emotional regulation, interpersonal functioning, and substance-use risk.

Her socioeconomic and gendered context is central to the formulation. B.A. lived on the streets of Addis Ababa for approximately three years, with no adult protection and extreme poverty. As a young woman in this context, she was at high risk for sexual exploitation, violence, coercion, and further trauma. The absence of effective social service intervention constitutes a form of structural neglect. Her clinical presentation, therefore, reflects not only individual trauma but also structural violence: homelessness, poverty, gender-based vulnerability, lack of child protection, and limited access to early mental health care.

Finally, B.A.’s lack of prior recognition of FDS reflects the diagnostic infrastructure limitations described in Section 2.5. Her symptoms were embedded in a broader system where seizure-like episodes are often interpreted through epilepsy, acute psychiatric crisis, substance use, or spiritual explanations rather than through a functional neurological framework. Consistent with the research and service priorities described in Section 2.5, her case underscores the need for culturally and clinically adapted FDS assessment pathways that integrate episode characteristics, trauma history, dissociation, suicidality and self-harm risk, substance-use screening, and socioeconomic vulnerability. Overall, B.A.’s episodes are best understood as involuntary dissociative responses to relational threat within a broader context of developmental trauma, abandonment, gendered vulnerability, and structural neglect.

#### 4.2.7 Treatment

Fluoxetine was initiated for moderate depressive symptoms. She engaged in individual and group therapy: psychoeducation, sleep hygiene, and grounding techniques; trauma-adapted CBT focused on emotion regulation and the rejection–episode link; trauma-focused work addressed sexual abuse and homelessness; group therapy supported relational skills; and motivational interviewing consolidated sobriety. Sessions occurred once to twice weekly.

#### 4.2.8 Outcome

Episode frequency fell from once or twice weekly to approximately once every two weeks by discharge. No self-harm or suicidal ideation occurred during her stay. She moved from guarded, somatic presentation toward greater engagement, verbalization of distress, and emotional regulation. She was referred to outpatient services. Given the chronicity of her trauma, prior polysubstance use, and brief placement, relapse risk remains significant, and coordinated aftercare is essential.

### 4.3 Case 3: M.S. | 23 years | female

#### 4.3.1 Presentation and episode pattern

M.S. was brought to the emergency department following a suicide attempt by ingestion of two strips of omeprazole within the center and was subsequently placed on active suicide watch. She reported recurrent episodes of loss of consciousness with involuntary limb movements, which she attributed to spirit possession, alongside persistent low mood and feelings of worthlessness. She described two prior suicide attempts over the past two years, both preceded by perceived rejection by her best friend, with a marked deterioration following the sudden loss of that relationship 18 months earlier. Episodes were typically preceded by hopelessness, worthlessness, and fears of abandonment. During events, she was unresponsive with eyes closed and had limited post-episode recall. The episodes began approximately 18 months prior to assessment, increased in frequency following admission, and occurred two to three times per week. There was no tongue biting, incontinence, prolonged postictal confusion, or injury. She also reported hearing voices exclusively in the peri-episodic period.

#### 4.3.2 Background and trauma history

M.S. was raised by an aunt; her parents lived in the countryside, and she described them as strangers. Her support narrowed after her aunt’s death and the loss of a close friendship. She relied heavily on religious interventions, including three months at a monastery and repeated holy-water treatment. Her socioeconomic situation was precarious, with occasional informal work, no stable housing, and no family safety net.

#### 4.3.3 Mental-status examination

She presented as appropriately dressed, calm, and cooperative. Speech was normal. Mood was depressed, and affect was blunted. Thought process was coherent; thought content was notable for suicidal ideation. She reported peri-episodic voices that were neither command-based nor persecutory; no visual hallucinations. She was fully oriented with intact cognition. Insight was partial.

#### 4.3.4 Standardize screening

ACE score: High risk.

GAD-7: Moderate.

PHQ-9: Moderate with suicide risk.

CAGE: Low risk.

SDS-Khat: No significant dependence.

#### 4.3.5 Examination and investigations

General physical and neurological examination did not reveal focal neurological deficits or signs of an acute medical illness. No blood, urine, or cerebrospinal-fluid testing, neuroimaging, or electroencephalography was performed; the assessment rested on history, directly observed episode semiology, and clinical examination, consistent with the resource constraints set out in Section 3.4. The seizure-like episodes preceded any pharmacotherapy; fluoxetine, a selective serotonin reuptake inhibitor, was started only afterwards as treatment, and no antipsychotic or antiepileptic medication was used.

#### 4.3.6 Culturally informed neuropsychiatric formulation

M.S. presents with a high-risk, complex neuropsychiatric picture in which clinically suspected functional/dissociative seizures (FDS) occur as part of a broader trauma-related and mood-related syndrome. Her seizure-like episodes occur in the context of emotional distress, particularly interpersonal loss, abandonment-related pain, and acute affective destabilization. The timing of symptom worsening after the sudden end of a close friendship suggests that relational loss functioned as a major precipitating stressor, activating longstanding abandonment themes connected to her estrangement from both parents.

Several observed features of the episodes support a clinically suspected FDS formulation. The episodes were associated with emotional distress, limited recall, absence of tonic-clonic motor activity, and absence of postictal confusion. These features are more consistent with functional or dissociative seizure-like episodes than with epileptic seizures. Although EEG evaluation was not available, the clinical pattern, emotional triggering, dissociative recall gaps, lack of a clear epileptic motor pattern, and absence of postictal confusion was less consistent with epilepsy and supports a clinically suspected FDS formulation.

M.S.’s suicide attempt by medication overdose places safety and stabilization at the center of the formulation. Her history of two suicide attempts within the previous two years, persistent low mood, worthlessness, limited social support, and escalating distress indicate clinically significant depressive and trauma-related burden. As discussed in Section 2.2, FDS is not a benign condition; it is associated with elevated mortality, psychiatric comorbidity, and suicide risk. In M.S.’s case, the seizure-like episodes should therefore not be viewed in isolation, but as part of a high-risk psychiatric presentation requiring suicide-risk management, emotional stabilization, and coordinated follow-up.

Her peri-episodic voice experiences require careful clinical interpretation. The reported voices occurred only before episodes, did not persist between episodes, had no command or persecutory content, and were not accompanied by formal thought disorder, sustained delusions, disorganized behavior, or functional deterioration consistent with a primary psychotic disorder. As discussed in Section 2.3, dissociation and trauma-related intrusions can mediate the relationship between traumatic experience and functional seizure-like episodes. In this context, M.S.’s voice-like experiences are best conceptualized as peri-episodic dissociative phenomena or trauma-related intrusions occurring at the threshold of episodes, rather than as psychotic hallucinations.

From the trauma framework discussed in Section 2.3, M.S.’s longstanding estrangement from both parents represents a major attachment injury. The sudden loss of a close friendship appears to have reactivated earlier abandonment-related pain, producing a level of loneliness, worthlessness, and emotional dysregulation that exceeded her available coping capacity. Her symptom timeline, mood deterioration, and seizure-like episodes beginning approximately 18 months before assessment and escalating toward overdose suggest cumulative decompensation rather than an isolated acute event.

From the neurobiological perspective outlined in Section 2.1, M.S.’s episodes can be understood as involuntary functional responses arising when emotional threat and relational loss overwhelm regulatory systems. Interpersonal loss may activate limbic threat and attachment-related circuits, while impaired top-down regulation may contribute to altered awareness, dissociative intrusions, and seizure-like expression. In this formulation, the episodes are real, involuntary, brain-based events shaped by trauma, emotional conditioning, and maladaptive threat prediction rather than conscious behavior.

From the dissociation framework discussed in Section 2.3, her limited recall of events and peri-episodic perceptual experiences suggests dissociative activation under conditions of affective overload. When grief, loneliness, and abandonment-related distress become intolerable, dissociative mechanisms may temporarily interrupt conscious awareness and convert emotional distress into bodily expression. This understanding helps distinguish her presentation from malingering, primary psychosis, or purely epileptic illness, while still recognizing the seriousness of her psychiatric risk.

Her attribution of the episodes to spirit possession should be understood in light of the cultural explanatory models discussed in Section 2.4. This belief is culturally embedded and should not be treated as evidence of delusional thinking. In the Ethiopian context, spirit-possession frameworks may provide meaning, reduce self-blame, and offer a pathway for help-seeking. However, exclusive reliance on religious interventions, including repeated holy water rituals, may delay psychiatric and neurological assessment when it becomes the only treatment pathway. A clinically appropriate approach should respect the meaning of her belief while introducing an additional neuropsychiatric explanation that links emotional distress, trauma-related arousal, dissociation, and functional seizure-like episodes.

M.S.’s socioeconomic and gendered vulnerability further intensifies the formulation. She had precarious informal work, no stable housing, minimal family support, and limited protection as a young single woman. These conditions increased her exposure to social shame, economic exploitation, and emotional isolation. Her lack of timely access to professional care reflects the diagnostic and service limitations described in Section 2.5, including limited access to neurological assessment, lack of video-EEG, and under-recognition of FDS in Ethiopian clinical settings.

Overall, M.S.’s presentation is best understood as clinically suspected FDS within a broader syndrome of depression, trauma-related distress, dissociation, abandonment-related vulnerability, and acute suicide risk. Her case illustrates the need, consistent with Section 2.5, for culturally adapted FDS assessment pathways that integrate seizure-like episode features, trauma history, dissociative symptoms, suicide risk, cultural explanatory models, and continuity of psychiatric care after discharge.

#### 4.3.7 Treatment

Following stabilization, M.S. remained on active suicide watch with close clinical monitoring. Fluoxetine was initiated, alongside psychoeducation linking emotional distress, trauma-related arousal, and seizure-like episodes. An integrative psychotherapeutic approach was implemented, combining supportive psychotherapy, trauma-focused cognitive behavioral therapy (CBT), and structured safety-focused interventions. These included safety planning, distress tolerance training, emotion regulation strategies, and identification of links between stressors, affective states, and episodes. Care was delivered in a culturally sensitive manner that did not directly confront or invalidate her explanatory beliefs regarding spirit possession. Sessions were conducted on a weekly basis, with ongoing suicide risk monitoring throughout the treatment period.

#### 4.3.8 Outcome

By her final month, she no longer reported active suicidal thoughts and showed improved emotional stability, engagement, and regulation. Episode frequency fell from two to three weekly to none in the final weeks. Some emotional vulnerability to interpersonal stress remained. She was referred for continued outpatient follow-up on graduation.

### 4.4 Case 4: B.Z. | 21 years | female

#### 4.4.1 Presentation and episode pattern

B.Z. presented with one year of recurrent loss of consciousness during which friends observed trembling and shaking of the arms and legs. Episodes were prolonged (up to 15 minutes) with immediate recovery; there was no postictal deep sleep, heavy breathing, tongue biting, incontinence, muscle aches, or prolonged confusion. She attributed the episodes to evil-spirit possession and had relied on holy-water rituals at various churches without prior medical evaluation. Episodes occurred two to three times weekly. She also reported difficulty concentrating during vocational training, difficulty forming relationships, and about one month of initial insomnia.

#### 4.4.2 Background and trauma history

B.Z. was raised in a rural area and entered a family-arranged marriage at age 12, during which she experienced sexual abuse. She remained in the marriage for approximately three years and had no children. After leaving the marriage, she relocated to Addis Ababa, where she worked as a maid. Her relationship with her family deteriorated, and she currently has no contact with them. Her background was one of rural poverty, early forced marriage, limited education, and financial instability.

#### 4.4.3 Mental-status examination

She was appropriately dressed and groomed, calm and cooperative, but initially guarded. Speech was normal and her mood was sad with restricted affect. Thought process was coherent; there was no suicidal ideation or delusional belief, and no hallucinations. She was fully oriented with intact memory. Insight was Level 3, and judgment was fair.

#### 4.4.4 Standardize screening

PHQ-9: Moderate.

GAD-7: Mild.

ACE score: High (with sexual abuse).

CAGE: Low risk.

SDS-Khat: Low risk.

#### 4.4.5 Examination and investigations

General physical and neurological examination did not reveal focal neurological deficits or signs of an acute medical illness. No blood, urine, or cerebrospinal-fluid testing, neuroimaging, or electroencephalography was performed; the assessment rested on history, directly observed episode semiology, and clinical examination, consistent with the resource constraints set out in Section 3.4. The seizure-like episodes preceded any pharmacotherapy; amitriptyline, a tricyclic antidepressant that can lower the seizure threshold, was prescribed only afterwards, at a low dose for insomnia, and no antipsychotic or antiepileptic medication was used.

#### 4.4.6 Culturally informed neuropsychiatric formulation

B.Z.’s episode characteristics are consistent with clinically suspected functional/dissociative seizures (FDS) within the broader framework of Functional Neurological Disorder. The episodes occurred in the context of emotional or interpersonal stress and were characterized by prolonged, variable motor activity, dissociative amnesia for the episode content, and an absence of postictal confusion. Episodes occurred approximately two to three times per week, with no neurological cause identified in the available clinical record. Although an EEG evaluation was not available at the referral center, the presentation should not be described as confirmed FDS. In this case, the clinical pattern was less consistent with epileptic seizures and more consistent with a functional or dissociative seizure-like presentation.

Her high ACE score and extensive trauma background place her within the vulnerability profile commonly described in the FDS literature. As discussed in Section 2.3, early developmental trauma, particularly childhood sexual abuse, is strongly associated with impaired emotional regulation, attachment disruption, dissociation, and vulnerability to functional seizure-like episodes. In B.Z.’s case, the trauma occurred within a coercive child-marriage context. Because she was 12 years old at the time, the marital arrangement cannot be considered a consensual adult marriage. Rather, it represented family-arranged child marriage in which sexual victimization occurred under the appearance of social legitimacy. This distinction is clinically important because the trauma was not only interpersonal but also structurally sanctioned by those responsible for her protection.

From the neurobiological perspective outlined in Section 2.1, early abuse and prolonged threat exposure may contribute to dysregulation of the hypothalamic-pituitary-adrenal axis and altered limbic-prefrontal connectivity, increasing vulnerability to involuntary functional neurological responses under stress. From the dissociation framework discussed in Section 2.3, her daily functioning may reflect an “apparently normal” self-state, while traumatic affect and memory remain held in dissociated emotional states that emerge during periods of interpersonal or emotional threat. Her guardedness, emotional numbing, attentional impairment, and dissociative recall gaps are therefore best understood as trauma-related neuropsychiatric features rather than evidence of malingering, psychosis, or an organic memory disorder.

B.Z.’s attribution of the episodes to spirit possession is culturally embedded and should be approached respectfully rather than dismissed. As discussed in Section 2.4, spirit-possession and evil-eye frameworks may provide culturally meaningful explanations for seizure-like episodes, but they can also delay neurological and psychiatric assessment when they become the exclusive treatment pathway. Prematurely imposing a purely biomedical explanation would likely undermine therapeutic trust and engagement. A more clinically appropriate approach is to validate the reality of her suffering while gradually introducing a neuropsychiatric explanation that links emotional distress, trauma-related arousal, dissociation, and functional seizure-like episodes.

Finally, her lack of timely access to mental health care reflects the structural barriers described in Section 2.5. Gender-based violence, poverty, disrupted education, family rejection, limited diagnostic infrastructure, and the absence of accessible trauma-informed care intersect to delay recognition and treatment. Her presentation, therefore, illustrates not only suspected FDS within a Functional Neurological Disorder framework but also the cumulative neuropsychiatric impact of developmental trauma, betrayal, structurally reinforced gender-based harm, cultural illness attribution, and systemic invisibility.

#### 4.4.7 Treatment

Early sessions established safety, trust, and rapport. Psychoeducation linked stress, emotion, trauma, and physical symptoms; grounding, emotion-regulation, and sleep-hygiene strategies were introduced. CBT principles explored the distress–episode relationship while respecting her spirit-possession belief, and trauma-focused work was introduced later, once she was comfortable discussing forced marriage, sexual abuse, and family separation. She also joined group therapy. Amitriptyline was prescribed for insomnia. Sessions were weekly.

#### 4.4.8 Outcome

Episode frequency fell markedly; by graduation, she was episode-free, compared with two to three episodes per week at admission. Concentration and social withdrawal improved but remained areas for continued work. She was referred to outpatient services. As elsewhere in the series, short placement and the absence of structured follow-up raise relapse risk; longer-term trauma-focused care and post-discharge contact would be needed to consolidate gains.

### 4.5 Case 5: B.S. | 24 years | female

#### 4.5.1 Presentation and episode pattern

B.S. presented with roughly 10 months of recurrent loss of consciousness with seizure-like activity. Observers noted irregular, asynchronous, asymmetric limb movements, prominent side-to-side head movements, and bilateral forced eye closure. Episodes lasted 5–15 minutes and ended abruptly, with rapid complete recovery and no postictal confusion, drowsiness, or headache. There was no tongue biting, incontinence, injury, or nocturnal occurrence. Episodes consistently occurred during or immediately after interpersonal conflict — most often arguments with family, friends, or a romantic partner — preceded by heightened emotional stress. She had experienced roughly 12–15 episodes in total, rising to two to three in the week before presentation during a breakup with her fiancé. She attributed her symptoms to spirit possession and sought repeated holy water rituals.

#### 4.5.2 Background and trauma history

She described a highly restricted upbringing, largely confined to the home with limited autonomy and peer contact; her social interaction was mostly with her brother. She believed these restrictions impaired her later capacity to form relationships and navigate social situations, while describing a generally positive relationship with her mother. She reported ongoing difficulty forming and maintaining friendships, challenges with interpersonal boundaries, and a tendency toward intense emotional investment, frequently resulting in conflict and rejection. A recent breakup appeared to heighten her emotional vulnerability and contributed to the increase in episodes. Her family was of lower income but not destitute; a restricted childhood likely reflected both poverty and cultural control over female children, and limited social skills may have contributed to difficulty in forming stable employment and relationships.

#### 4.5.3 Mental-status examination

She presented as well-groomed, intentionally styled, and cooperative, with somewhat accelerated speech. She described her mood as “fine,” though affect was labile, reactive, and at times exaggerated. Thought process was coherent; thought content showed heightened sensitivity to relationships and a tendency to perceive closeness more intensely than reciprocated. No delusions or perceptual disturbances were elicited; cognition was intact. Insight was partial.

#### 4.5.4 Standardize screening

PHQ-9: Low risk.

GAD-7: Low risk.

ACE score: Intermediate.

CAGE: Low risk.

SDS-Khat: Low risk.

#### 4.5.5 Examination and investigations

General physical and neurological examination did not reveal focal neurological deficits or signs of an acute medical illness. No blood, urine, or cerebrospinal-fluid testing, neuroimaging, or electroencephalography was performed; the assessment rested on history, directly observed episode semiology, and clinical examination, consistent with the resource constraints set out in Section 3.4. No antidepressant, antipsychotic, or antiepileptic medication was prescribed; management was psychological.

#### 4.5.6 Culturally informed neuropsychiatric formulation

B.S.’s clinical presentation is consistent with clinically suspected functional/dissociative seizures (FDS) within the broader framework of Functional Neurological Disorder. The episodes are closely linked to acute interpersonal stress, emotional dysregulation, and perceived relational threat. Across the reported episodes, the most consistent precipitating factor was interpersonal conflict, particularly arguments with family members, friends, or romantic partners. This pattern suggests that the seizure-like events functioned as involuntary neuropsychiatric responses to emotional states that exceeded her available coping capacity, rather than as episodes arising from epileptiform brain activity.

Several semiological features support the FDS formulation. The episodes were characterized by irregular and asynchronous limb movements, prominent side-to-side head movements, bilateral forced eye closure, preserved or fluctuating awareness, and rapid recovery without postictal confusion, drowsiness, or headache. The absence of tongue biting, urinary or fecal incontinence, significant injury, nocturnal events, and prolonged postictal recovery further reduces the likelihood of generalized tonic-clonic epileptic seizures. Although EEG evaluation was not available at the referral center, the overall clinical pattern — especially the clear association with emotional triggers, non-stereotyped motor activity, fluctuating responsiveness, and rapid return to baseline — was less consistent with epileptic seizures and more consistent with functional or dissociative seizure-like episodes.

B.S.’s developmental history provides an important vulnerability pathway. Her highly restricted childhood environment appears to have limited normal peer socialization and the development of interpersonal skills. She reported minimal exposure to relationships outside the home and described longstanding difficulty forming and maintaining friendships. These developmental restrictions likely contributed to difficulty recognizing interpersonal boundaries, emotional dependency, difficulty tolerating relational distance, and heightened sensitivity to perceived rejection or interpersonal disconnection. In this context, conflict may have been experienced not simply as disagreement but as a threat to attachment security, belonging, and emotional stability.

On evaluation, B.S. met DSM-5-TR criteria for histrionic personality disorder. We report this diagnosis with explicit caution: in a young, acutely stressed woman with a restricted developmental history, personality-disorder labels risk pathologizing trauma-shaped relational patterns, may be unstable over time, and should be held provisionally rather than as a fixed explanatory endpoint. The finding provides additional clinical context rather than a moral judgment or an explanation of intentional behavior. As discussed in Section 2.3, FDS vulnerability is often shaped by early adversity, affective dysregulation, destabilizing interpersonal patterns, and personality-related features. In B.S.’s case, histrionic personality features likely interacted with her restricted developmental history and limited social learning, increasing vulnerability to intense emotional escalation during conflict. Her tendency to become overly involved or intense in relationships may have contributed to repeated interpersonal strain, which then became a recurring trigger for dissociative or functional episodes.

From the neurobiological perspective outlined in Section 2.1, her episodes can be understood as involuntary functional responses arising when emotional arousal overwhelms cognitive regulation. Interpersonal conflict may activate limbic threat systems, while reduced top-down regulatory control may contribute to loss of voluntary control, altered awareness, and motor expression through non-epileptic seizure-like activity. The patient’s report that she could sometimes hear voices but was unable to respond voluntarily is consistent with fluctuating awareness and dissociative detachment rather than a primary psychotic process. There was no evidence of persistent hallucinations, formal thought disorder, delusional beliefs beyond the cultural explanatory framework, or functional decline suggestive of a primary psychotic disorder.

From the dissociation and psychiatric-comorbidity framework discussed in Section 2.3, the episodes may represent a functional “shutdown” or dissociative response during states of interpersonal overload. Her emotional system appears to become activated rapidly during conflict, particularly when she perceives rejection, misunderstanding, or relational instability. When emotional arousal exceeds her capacity for reflective regulation, the body appears to express distress through altered responsiveness and non-stereotyped motor behavior. This pattern is consistent with the understanding of FDS as an embodied expression of emotional conflict and dysregulated threat processing, rather than conscious symptom production.

Her attribution of the episodes to evil spirit possession is culturally embedded and should be understood within the explanatory models discussed in Section 2.4. This belief does not, by itself, indicate psychosis. Rather, it reflects a culturally available framework for explaining seizure-like episodes, especially in settings where mental health literacy is limited and functional neurological symptoms are poorly recognized. However, repeated reliance on holy water rituals and religious interventions may have contributed to delayed clinical assessment and reinforced the belief that the episodes were caused exclusively by external spiritual forces. A respectful clinical approach should therefore validate the reality of her symptoms while gradually introducing a neuropsychiatric explanation that links interpersonal stress, emotional arousal, dissociation, and functional seizure-like episodes.

Finally, the absence of video-EEG and the lack of an established FDS diagnostic pathway reflect the limitations of the diagnostic infrastructure described in Section 2.5. Her restricted childhood socialization also reflects the broader gendered and socioeconomic context that shaped her vulnerability, including culturally reinforced control over female children, limited autonomy, and later economic dependence. Her case illustrates a pattern of FDS vulnerability not primarily associated with a single catastrophic trauma, but with cumulative developmental restriction, limited relational development, gendered social control, interpersonal dysregulation, cultural illness attribution, and limited access to early psychological support. The broader need for culturally adapted assessment and systematic Ethiopian FDS research is consistent with the priorities described in Section 2.5.

#### 4.5.7 Treatment

The diagnosis was communicated supportively, emphasizing that the episodes are real, brain-based, and not intentionally produced, and aligning the explanation with her belief system while gently introducing a biopsychosocial understanding. An integrated approach combined CBT, trauma-informed principles, and interpersonal work. Early sessions used psychoeducation about Functional Neurological Disorder and structured trigger monitoring. Cognitive restructuring addressed beliefs about rejection and abandonment; grounding and emotion-regulation skills (sensory grounding, paced breathing, present-moment orientation) targeted dissociative escalation. Interpersonal interventions addressed boundaries, relationship intensity, and social-cue interpretation through role-play and rehearsal. No anti-epileptic medication was prescribed; psychological intervention was prioritized.

#### 4.5.8 Outcome

Over four months, she reported a marked reduction in episodes from a peak of two to three weekly to only two in the preceding month with shorter, less distressing events, improved trigger awareness, and better interpersonal functioning. Continued therapy was recommended to consolidate gains and address underlying relational dynamics.

### 4.6 Case 6: R.H. | 19 years | female

#### 4.6.1 Presentation and episode pattern

R.H. presented with marked emotional distress, poor sleep, persistent sadness, hopelessness, limited eye contact, and anxious mood, with intermittent khat use. She reported recurrent nightmares involving her child that severely disrupted sleep; after admission, she deteriorated following such nightmares and attempted suicide, requiring transfer for inpatient care. She also had recurrent loss of consciousness beginning about nine years earlier and worsening after separation from her child; prior visits to health centers had not led to long-term treatment. Episodes occurred mainly during emotional dysregulation, preceded by intense anxiety, persistent thoughts of her child, and dizziness; observers described sudden loss of consciousness with irregular limb movements and prolonged duration, with limited recall and subsequent fatigue, headache, and emotional upset. There was no tongue biting, incontinence, injury, or nocturnal occurrence. She attributed the episodes to “Zar” or “azurit” and reported frequent sudden falls.

#### 4.6.2 Background and trauma history

R.H. has a three-year-old child who has lived with relatives in Jimma for about two years. She explained that, because of her frequent episodes, an uncle took the child away, although she has no clear memory of the circumstances. Since the separation, she has had intrusive thoughts and frequent frightening nightmares involving her child, with persistent sadness, guilt, helplessness, poor sleep, anxiety, irritability, and difficulty concentrating. She reported sexual abuse at age 14 but declined to provide further details. Her situation was extremely precarious, with no independent income and reliance on extended family increasingly unwilling to support her; as a young mother without custody, she faced severe social shame and marginalization.

#### 4.6.3 Mental-status examination

She was cooperative with limited eye contact. Speech was slow. Mood was anxious and affect restricted. Thought process showed flight of ideas; thought content included suicidal thoughts. No hallucinations were reported. She was oriented to person, place, and time, with impaired attention and concentration. Insight appeared good.

#### 4.6.4 Standardize screening

ACE score: High (with significant sexual abuse).

GAD-7: Mild.

PHQ-9: Moderate with suicide risk identified.

CAGE: Low risk.

SDS-Khat: No significant dependence.

#### 4.6.5 Examination and investigations

General physical and neurological examination did not reveal focal neurological deficits or signs of an acute medical illness. No blood, urine, or cerebrospinal-fluid testing, neuroimaging, or electroencephalography was performed; the assessment rested on history, directly observed episode semiology, and clinical examination, consistent with the resource constraints set out in Section 3.4. The seizure-like episodes had been present for years before any pharmacotherapy; because of the longstanding history and an earlier concern about epilepsy, phenobarbital 100 mg daily was started empirically at presentation and then tapered as the picture became characteristic of FDS, and fluoxetine 20 mg daily was added for depressive and anxiety symptoms. No antipsychotic medication was used.

#### 4.6.6 Culturally informed neuropsychiatric formulation

The clinical picture is consistent with clinically suspected functional/dissociative seizures (FDS) within the framework of Functional Neurological Disorder rather than epilepsy. The seizure-like episodes were strongly associated with emotional distress and interpersonal triggers, particularly thoughts and nightmares involving separation from her child. The episodes showed prolonged and irregular motor activity rather than stereotyped rhythmic movements typically seen in epileptic seizures. There was no history of tongue biting, urinary incontinence, significant injury, nocturnal episodes, or postictal confusion. Instead, she described emotional exhaustion following the episodes. The episodes also appeared closely linked to unresolved trauma, grief, anxiety, and psychosocial stressors, further supporting a clinically suspected functional neurological formulation.

Several observed features of the episodes support the FDS formulation. The events involved sudden loss of responsiveness, irregular limb movements, prolonged duration, limited recollection, and emotional exhaustion afterward, rather than postictal confusion. There was no history of tongue biting, urinary incontinence, significant injury, nocturnal episodes, or stereotyped rhythmic motor activity typical of generalized tonic-clonic epileptic seizures. Although EEG evaluation was not available, the available clinical episode features, especially emotional triggering, prolonged irregular movements, dissociative recall gaps, and lack of postictal confusion, were more consistent with functional or dissociative seizure-like episodes than epilepsy.

R.H.’s psychiatric presentation was clinically acute and high risk. She reported poor sleep, persistent sadness, hopelessness, anxious mood, recurrent nightmares involving her child, and a suicide attempt following worsening distress. Her PHQ-9 showed moderate depressive symptoms with suicide risk identified, while the GAD-7 indicated mild anxiety symptoms. As discussed in Section 2.2, FDS is associated with elevated psychiatric morbidity and increased mortality, particularly when depression, trauma, self-harm, and suicidality are present. In R.H.’s case, suicide risk is not secondary or incidental; it is central to the formulation and requires stabilization, safety planning, psychiatric monitoring, and continuity of care.

Her trauma history provides a major vulnerability pathway. R.H. reported sexual abuse at age 14, a high ACE score, and prolonged separation from her three-year-old child. As discussed in Section 2.3, childhood sexual abuse and early adversity increase vulnerability to FDS through disruption of emotional regulation, attachment security, self-concept, and threat processing. In R.H.’s case, the sexual abuse likely contributed to trauma-related emotional dysregulation, while the separation from her child appears to function as an ongoing attachment wound and major maintaining stressor. The recurrence of nightmares and seizure-like episodes involving her child suggests that unresolved grief and maternal separation may be contributing factors to both her emotional decompensation and functional neurological symptoms.

From the dissociation and psychiatric-comorbidity framework discussed in Section 2.3, R.H.’s limited recollection of episodes, emotional exhaustion after events, and symptom worsening during nightmares are consistent with dissociative activation under conditions of trauma-related arousal. Her episodes appear to occur when grief, fear, and helplessness become psychologically intolerable. Rather than producing deliberate behavior, the nervous system appears to shift into a functional shutdown or dissociative state, with altered responsiveness and irregular motor activity. This formulation is further supported by the absence of postictal confusion and the close temporal link between emotional triggers and episodes.

From the neurobiological perspective outlined in Section 2.1, R.H.’s episodes can be understood as involuntary functional responses arising when trauma-related and attachment-related threat systems overwhelm cognitive regulation. Reminders of her child, especially nightmares and intrusive thoughts, may activate limbic threat circuits and interoceptive arousal. When top-down regulatory control is insufficient, emotional distress may be expressed through altered awareness and non-epileptic motor activity. In this framework, the episodes are real, brain-based, and involuntary, but they are generated through functional neuropsychiatric mechanisms rather than epileptiform electrical activity.

Intermittent khat use is clinically relevant to the formulation. Although no other substance use was reported, khat has stimulant properties and may worsen anxiety, sleep disturbance, irritability, physiological arousal, and emotional instability. In R.H.’s case, khat use may have contributed to sleep disruption and increased vulnerability to affective escalation, particularly in the context of nightmares, depressive symptoms, and trauma-related distress. Accordingly, it should be considered clinically as a potential maintaining or exacerbating factor, while ensuring that the presentation is not reduced to substance use alone.

R.H.’s attribution of her episodes to “Zar” or “azurit” should be understood within the cultural explanatory models discussed in Section 2.4. This attribution does not indicate psychosis. Rather, it reflects a culturally available framework for explaining sudden falls, altered responsiveness, and seizure-like episodes. At the same time, when spiritual explanations become the only pathway for understanding and treatment, they may delay psychiatric and neurological assessment. A respectful clinical approach should validate the reality and cultural meaning of her experience while gradually introducing a neuropsychiatric formulation linking trauma, grief, nightmares, emotional arousal, dissociation, and functional seizure-like episodes.

R.H.’s socioeconomic and gendered context is also central. She had no independent income, depended on extended family, and faced increasing rejection because of her episodes. As a young mother separated from her child, she also faced severe stigma and marginalization. The removal of her child by relatives may have been intended as protection, but it also appears to have intensified her grief, helplessness, and psychological instability. This creates a painful reinforcing cycle: seizure-like episodes contributed to separation from her child, and separation from her child then became a major trigger for nightmares, emotional collapse, suicidality, and further episodes.

Finally, R.H.’s lack of a clear prior diagnosis and limited access to specialized neurological evaluation reflect the diagnostic infrastructure barriers described in Section 2.5. Her presentation illustrates how suspected FDS may persist for years in Ethiopia in the absence of video-EEG confirmation, limited recognition of functional neurological symptoms, and the predominance of culturally framed explanatory models in help-seeking pathways. Consistent with the priorities outlined in Section 2.5, her case underscores the need for culturally adapted FDS assessment pathways that integrate seizure-like episode features, trauma history, maternal separation, dissociation, mood symptoms, suicidality, substance-use screening, and continuity of care after discharge. Overall, R.H.’s presentation may be understood as suspected FDS occurring within a complex clinical context characterized by unresolved grief, trauma-related distress, depressive symptoms, dissociative vulnerability, stimulant-related arousal, and structural social marginalization.

#### 4.6.7 Treatment

Fluoxetine 20 mg daily was initiated for depressive and anxiety symptoms. Phenobarbital 100 mg daily was started initially, given the longstanding episodes and prior epilepsy concern, but as the picture became increasingly suggestive of FDS, gradual discontinuation was planned. Psychotherapy was integrative and flexible: supportive work emphasized containment, distress tolerance, and alliance; psychoeducation addressed trauma reactions and the functional nature of episodes; CBT elements targeted maladaptive thoughts and catastrophic thinking; grounding supported stabilization. Sessions addressed grief over child separation, emotion regulation, sleep, suicide-risk management, and stress. She was closely monitored for mood, suicidality, and episode frequency.

#### 4.6.8 Outcome

There was some improvement in depressive symptoms and engagement, with better communication and eye contact, and a degree of reduction in episode frequency. However, significant emotional vulnerability remained, particularly around nightmares and intrusive thoughts of her child. Following one such episode of worsening, she attempted suicide with aggravation of symptoms. Given persistent symptoms, ongoing suicide risk, and unresolved trauma, she was discharged with a recommendation for further psychiatric stabilization before enrollment into vocational training.

## 5 Thematic analysis

The six de-identified case narratives were analyzed using the framework-based thematic analysis described in Section 3.5, organized around the conceptual model in Section 2. The unit of analysis was the complete clinical narrative. The coding framework is summarized in **Table 2**; the five resulting themes are mapped in **Figure 3**, and their cross-case distribution is set out in **Table 3**. The analysis is interpretive and clinically grounded, and prevalence statements describe this purposive sample only rather than providing an epidemiological estimate.

Across the six cases, five interrelated themes were identified: (1) neurological episode features consistent with suspected functional or dissociative seizures; (2) betrayal, abandonment, and attachment disruption as vulnerability pathways; (3) dissociation and functional shutdown under affective overload; (4) psychiatric risk, personality organization, and comorbidity; and (5) culture, gendered vulnerability, and systemic invisibility. The themes are presented separately for clarity but are clinically intertwined in every case, as shown in the thematic map (**Figure 3**): each theme is anchored to the central phenomenon, while the dashed connections indicate how the themes acted on one another within individual patients.

### 5.1 Theme 1: neurological episode features consistent with suspected functional or dissociative seizures

All six cases featured seizure-like episodes whose clinical characteristics were more consistent with suspected FDS than with epilepsy: prolonged duration, irregular or asynchronous movements, altered responsiveness, preserved or fluctuating awareness, limited recall, absence of postictal confusion, and close association with emotional or interpersonal triggers. Several cases additionally showed forced eye closure, side-to-side head movements, sudden falls, sensory prodromes, or post-episode emotional exhaustion. These positive features are analytically central: in the absence of any video-EEG, they constitute the principal evidence on which the functional formulation rests, and they are reported here not as a checklist applied retrospectively but as features that trained residential staff directly observed across repeated episodes.

The diagnostic reasoning was as much about what was absent as about what was present. None of the cases showed the strongly epilepsy-suggestive features that the pathway in **Figure 2** screens for: stereotyped brief automatisms, tongue biting, urinary or fecal incontinence, nocturnal occurrence, injury, or prolonged postictal confusion. Instead, recovery was typically rapid and followed by emotional discharge (crying, screaming, agitation in B.A. and M.S.) or fatigue and tearfulness (E.A., R.H.), rather than the dense postictal somnolence and disorientation characteristic of generalized seizures. This combination of present positive features and absent epileptic features is precisely the configuration that the staged approach treats as supporting a suspected functional diagnosis where EEG is unavailable (Section 3.4).

Importantly, the episodes were not uniform, and the heterogeneity is informative in itself. E.A. had a reproducible interoceptive prodrome (burning ear pain, tinnitus); B.S. showed the most florid motor semiology, with irregular asynchronous limb movements, prominent side-to-side head movement, and bilateral forced eye closure; R.H. had prolonged irregular movements with marked emotional exhaustion; M.S. had unresponsiveness with eye closure and limited recall but no convulsive activity; B.A. showed minimal motor activity with an approximately ten-minute gap in awareness; and B.Z. had prolonged, variable movement with dissociative recall gaps. This variability across patients, combined with within-patient dependence of episodes on emotional and relational context, is more consistent with a functional mechanism than with a single stereotyped epileptic focus. Consistent with the neurobiological model in Section 2.1, the episodes are best understood as involuntary, brain-based events in which altered emotion regulation, interoception, motor control, and threat processing are expressed through the body — not as feigned behavior, purely psychological narratives, or culturally produced performances.

### 5.2 Theme 2: betrayal, abandonment, and attachment disruption as vulnerability pathways

All six cases involved clinically meaningful attachment disruption, relational deprivation, or developmental betrayal: parental abandonment and prolonged loss of contact (E.A.); maternal rejection, sexual abuse, and years of homelessness (B.A.); estrangement from both parents compounded by the sudden loss of a sustaining friendship (M.S.); family-arranged child marriage with sexual abuse and subsequent family estrangement (B.Z.); a highly restricted childhood that impaired later relational capacity (B.S.); and sexual abuse together with forced separation from her own child (R.H.). Vulnerability here was not reducible to a single discrete trauma but reflected cumulative developmental disruption — attachment injury, abandonment, gendered coercion, and chronic relational insecurity — consistent with the trauma-and-attachment pathway outlined in Section 2.3.

A consistent clinical pattern was the reactivation of early attachment injuries by present-day relational events, so that the same history functioned as both a predisposing and a precipitating factor. This sequence was clearest where an identifiable interpersonal trigger immediately preceded escalation: B.A. ‘s episodes intensified after a phone call in which her mother told her never to make contact again; M.S. deteriorated after the abrupt loss of her closest friendship and following perceived rejection; R.H. ‘s episodes and nightmares centered specifically on separation from her child; and B.S.’s episodes clustered around conflict and a breakup. Within an attachment framework, these events were not experienced as ordinary disappointments but as confirmations of abandonment, mobilizing fear, shame, and helplessness at an intensity that overwhelmed the patient’s capacity to regulate affect.

This theme has direct therapeutic implications. Because relational threat was the most reliable proximal trigger across the series, interventions that addressed only the episodes — without attending to rejection sensitivity, grief, and the meaning of attachment loss — would be unlikely to produce durable change. The cases in which therapy explicitly targeted abandonment and relational dynamics (E.A., B.A., M.S., B.S.) showed the clearest reductions in episode frequency, whereas R.H., whose central attachment wound (loss of custody) remained unresolved and structurally addressable during her stay, retained the greatest vulnerability. The theme, therefore, supports an attachment-informed, trauma-focused model of care rather than symptom suppression alone.

### 5.3 Theme 3: dissociation and functional shutdown under affective overload

Dissociation was a central mechanism across the series and provided the clearest bridge between psychological distress and the physical episodes. Patients consistently described recall gaps, altered responsiveness, emotional overwhelm immediately preceding loss of awareness, peri-episodic perceptual experiences, and an inability to respond voluntarily despite sometimes retaining partial awareness: emotional overwhelm followed by an approximately ten-minute loss of awareness (B.A.); limited recall accompanied by peri-episodic voices (M.S.); dissociative gaps for episode content (B.Z.); fluctuating awareness with voices heard while unable to respond (B.S.); limited recall with subsequent exhaustion (R.H.); and difficulty recalling episode detail (E.A.).

Consistent with Section 2.3, these phenomena are best understood as functional shutdown states that arise when affective and threat-related arousal exceeds the capacity for regulatory control, at which point dissociative processes interrupt conscious awareness and convert emotional distress into bodily expression. The model accounts for the observed sequence — trigger, rapid affective escalation, interoceptive or perceptual prodrome, loss of voluntary responsiveness, and post-episode depletion — without invoking either conscious simulation or epileptiform discharge.

Clinically, the most consequential aspect of this theme is its bearing on the differential diagnosis with primary psychosis. The peri-episodic voice-like experiences reported by M.S. and B.S. were tightly bound to the episodes themselves: they occurred only at the threshold of an episode, did not persist in the inter-episode period, lacked command or persecutory content, and were unaccompanied by thought disorder, sustained delusions, negative symptoms, or functional decline. Interpreted within a dissociation framework, they are trauma-related intrusions rather than psychotic hallucinations. This distinction is not academic. Misreading dissociative intrusions as psychosis would risk inappropriate antipsychotic exposure, reinforce stigma, and in this cultural context it may intensify spirit-possession attributions and increase reliance on holy-water or exorcism pathways, further delaying trauma-focused care. Recognizing dissociation as the operative mechanism reframes the episodes as treatable functional phenomena and directs treatment toward grounding, affect regulation, and trauma processing.

### 5.4 Theme 4: psychiatric risk, personality organization, and comorbidity

The series carried a substantial psychiatric burden that extended well beyond the episodes themselves, underscoring that FDS in this population cannot be reduced to an isolated seizure-like symptom. M.S. and R.H. presented with acute suicidality and recent attempts; R.H. additionally showed moderate depressive symptoms, recurrent nightmares, sleep disruption, and intermittent khat use. B.A. had a history of repeated self-harm by cutting and marked rejection sensitivity. B.Z. showed moderate depressive features, emotional numbing, and trauma-related attentional impairment. E.A. presented with low mood, restricted affect, and mild anxiety. Consistent with Sections 2.2 and 2.3, the episodes occurred within broader neuropsychiatric profiles in which depression, anxiety, trauma related symptoms, and critically suicide risk were frequently the more dangerous clinical problems.

Risk was unevenly distributed, and the analysis highlights the importance of stratification over uniform management. M.S., who was assessed following an in-facility overdose and had two prior attempts, and R.H., who attempted suicide during her admission after a nightmare relating to her child, required urgent, safety-centered care; for both, suicide-risk management was prioritized over seizure-directed intervention. Others (E.A., B.S.) carried comparatively lower acute risk. This distribution argues for routine, structured suicide-risk and self-harm assessment as a non-negotiable component of FDS evaluation in such settings (Step 3 in **Figure 2**), and for treatment plans that integrate psychotherapy with management of comorbid depression and anxiety, trauma-focused interventions, and substance use screening.

The personality-related findings warrant particular interpretive caution. B.S. met DSM-5-TR criteria for histrionic personality disorder, and B.A. showed borderline-range relational features, but in young, acutely traumatized women with restricted developmental histories, such presentations may equally reflect complex post-traumatic adaptations, and personality labels risk pathologizing trauma-shaped relational patterns that may not be stable over time. As stated in the case formulations (Sections 4.2, 4.5), these diagnoses are reported provisionally and descriptively rather than as fixed explanatory endpoints; their main clinical value here is in guiding interpersonally informed therapy, not in reclassifying the seizure phenomenon.

### 5.5 Theme 5: culture, gendered vulnerability, and systemic invisibility

Cultural explanatory models shaped how every patient understood her episodes and sought help: the evil eye (E.A.); spirit possession (M.S., B.Z., B.S.); and Zar or azurit (R.H.). Consistent with Section 2.4, these frameworks are locally coherent rather than delusional and may reduce self-blame and confer meaning, particularly where psychiatric explanations carry stigma. The clinical issue is not the belief itself but the consequences of exclusive reliance on it: several patients had pursued holy-water rituals, monastery stays, or church-based interventions. In some cases, the only prior response to years of episodes likely delayed assessment, reinforced fear, deepened dependency on non-medical pathways, and, in some instances, imposed financial and social costs. A culturally responsive stance, therefore, validates each patient’s explanatory model and works alongside it, while gradually introducing a neuropsychiatric understanding rather than confronting or dismissing the cultural account.

Gendered vulnerability was equally central and, in this series, inseparable from the clinical picture. The cases involved young women whose symptoms emerged in the context of abandonment, sexual abuse, family-arranged child marriage, homelessness, restricted female autonomy, economic dependency, family rejection, and forced separation from a child. Several of these are not merely background stressors but structurally sanctioned, gendered harms: B.Z.’s marriage at age 12 placed sexual victimization under the appearance of social legitimacy; B.A.’s years of street homelessness exposed her to exploitation without protective services; R.H.’s loss of custody produced profound grief and social shame; and B.S.’s restricted upbringing reflected gendered control over young women’s autonomy. As discussed in Section 2.5, these conditions functioned as clinical variables, shaping both vulnerability to the disorder and access to care, rather than simply as social context.

The result was a systemic invisibility across all cases: none of the six had ever received a FDS diagnosis, no EEG was available, and several had encountered only religious care or no clear pathway at all despite years of disabling episodes. A treatable functional neurological condition was thus repeatedly unnamed, unmeasured, and unmanaged. This theme reframes the case series as evidence of a service-level gap rather than only a set of individual presentations, and it identifies the concrete targets for change: culturally adapted, EEG-independent assessment pathways; integration of trauma-informed and gender-sensitive care; and the establishment of referral and continuity mechanisms so that the recognition achieved during a residential stay is not lost on discharge.

### 5.6 Summary of thematic findings

The six cases revealed a consistent neuropsychiatric and sociocultural pattern. Suspected functional/dissociative seizures (FDS) were supported not only by histories of trauma and psychological distress but also by positive clinical features — altered responsiveness, irregular motor activity, fluctuating awareness, dissociative recall gaps, absence of postictal confusion, and lack of strong epileptic features. These findings align with the neurobiological framework of FDS, and the staged diagnostic approach discussed earlier. Across cases, episodes occurred within contexts characterized by developmental trauma, attachment disruption, dissociation, psychiatric comorbidity, gender-based vulnerability, cultural interpretations of illness, and limited diagnostic recognition. The presentation is neither purely neurological nor purely psychological: it represents an embodied, culturally mediated, and structurally shaped neuropsychiatric condition. Collectively, these findings support the central argument of this study: effective assessment and management of FDS in Ethiopia require an integrated framework that recognizes functional neurological symptoms, evaluates trauma and psychiatric risk, incorporates cultural understandings, minimizes diagnostic delay, and promotes continuity of care.

## 6 Discussion

This case series provides preliminary evidence that clinically suspected FDS occurred in young women receiving care at a single Ethiopian rehabilitation center. The cases align with the broader FDS literature, which commonly reports a predominance of young women, high rates of childhood adversity and psychiatric comorbidity, and dissociative and functional neurological symptom features (3, 14). Because no EEG was available, the cases are best described as clinically suspected rather than confirmed FDS (Section 3.4).

The findings support a combined neuropsychiatric and sociocultural formulation. Episode features were consistent with functional or dissociative seizures, while trauma, attachment disruption, altered threat processing, dissociation, emotional dysregulation, gendered vulnerability, poverty, cultural meaning-making, and diagnostic scarcity shaped onset, maintenance, help-seeking, and treatment response. This integration is consistent with international calls to treat functional neurological disorder as a positively diagnosed, treatable condition rather than a diagnosis of exclusion or an expression of feigning and malingering (8, 31).

The clinical and rehabilitation implications are substantial. Patients showed improvement when care integrated non-stigmatizing psychoeducation, CBT-informed strategies, and trauma-focused stabilization, alongside supportive psychotherapy, interpersonal skills training, psychiatric risk management, selective use of psychotropic medication for comorbid depression, and structured referral planning. This multimodal approach aligns with the established evidence base for psychological therapies in dissociative seizures (32, 33). However, improvement was often fragile: post-discharge continuity was limited, and many social stressors persisted, suggesting that gains achieved in a protected residential setting may not be sustained after return to adversity.

Cultural and religious explanations should be approached respectfully but should not substitute for evidence-based assessment and treatment. Zar, Buda, Azurit, evil eye, spirit possession, holy water, and exorcism may provide meaning, social support, and culturally coherent explanations for distress; however, exclusive reliance on such approaches can delay care, reinforce fear and stigma, increase financial burden, and expose vulnerable individuals to harmful practices (5, 6, 34). A culturally responsive model validates the patient’s explanatory framework while gradually introducing a neuropsychiatric account. The broader contribution of this series is to begin to make an under-described presentation of functional/dissociative seizures in this Ethiopian rehabilitation context visible and to offer a pragmatic, EEG-independent assessment pathway (**Figure 2**) whose transferability would need to be tested in comparable low-resource settings.

Several interpretive cautions follow from the design. The reductions in episode frequency reported here were observed during and shortly after a period of intensive residential care; they are uncontrolled clinical observations and should not be read as proof of treatment efficacy or as evidence of a causal mechanism. There was no comparison group, follow-up was short, and improvement could reflect the protected residential environment, regression to the mean, natural fluctuation in symptoms, or non-specific therapeutic factors rather than any specific intervention. Equally, the observation that episodes lessened with psychological treatment is not in itself diagnostic of functional/dissociative seizures, because response to such care does not reliably distinguish them from epilepsy or other conditions. We therefore avoid causal and efficacy language in describing these outcomes, and restate these constraints in the Limitations (Section 7).

The cultural dimension of these cases warrants closer examination, because culture did not merely surround the episodes but shaped their expression, interpretation, and clinical course. In this series, idioms such as Zar, Buda, azurit, and the evil eye supplied the explanatory vocabulary through which patients and families understood altered consciousness and involuntary movement, and they directed help-seeking toward holy water, monastery stays, prayer, and exorcism before, and often instead of, biomedical care. These attributions were not incidental: they influenced symptom expression (for example, peri-episodic perceptual phenomena interpreted as spirit communication), the timing and route of presentation, the degree of family involvement, and the stigma attached to a psychiatric formulation. The Ethiopian context also differs in important respects from the settings where most functional-seizure research has been conducted. Compared with high-income North American, European, and Australian health systems—and even with some other African and Middle Eastern settings—Ethiopia combines a particularly severe scarcity of neurological and EEG services (Section 2.5), a strong and institutionally embedded religious-healing sector, gendered constraints on young women’s autonomy and help-seeking, and specific possession and evil-eye idioms that are distinct from, though analogous to, explanatory models described elsewhere. Cultural explanatory models therefore cannot be treated as interchangeable across regions, and findings from better-resourced settings may not transfer directly to this one.

Recognizing these models has clear advantages rather than being merely an obstacle to be overcome. Culturally grounded explanations can reduce self-blame, mobilize family and community support, offer a coherent narrative for frightening experiences, and provide an existing, trusted pathway of care; dismissing them outright risks alienating patients and deepening stigma. A culturally informed approach can therefore build deliberate bridges between these frameworks and evidence-based psychological treatment. Cognitive behavioural and trauma-focused techniques can be reframed in locally meaningful terms—for example, mapping the experience of an overwhelming spiritual or emotional “attack” onto a psychoeducational account of threat arousal and limbic-motor dysregulation, validating the reality and seriousness of the experience while gradually reattributing it to a treatable mind–body process, and engaging respected religious or community figures as collaborators rather than competitors in care. Practically, this implies collaborative formulation that names the patient’s own explanatory model, psychoeducation delivered in cultural idiom, coordination with faith-based caregivers, attention to gendered safety and structural barriers to follow-up, and the use of brief, transferable assessment pathways (**Figure 2**) where specialist services are inaccessible. These suggestions are hypotheses generated by a small case series and would require evaluation in larger, prospective, and ideally controlled studies.

## 7 Limitations

The most significant limitation is the absence of EEG data; epilepsy and mixed seizure disorders cannot be definitively excluded, and the formal LaFrance diagnostic level is therefore “possible.” The clinical formulations, therefore, rely on observed episode features, psychiatric evaluation, trauma history, cultural context, and clinical course (Section 3.4). The sample is small and drawn from a single residential rehabilitation center in Addis Ababa, so the findings are not generalizable to all Ethiopian patients with seizure-like episodes. The analysis is retrospective and based on clinical documentation rather than prospective research interviews, which constrains the depth and consistency of thematic interpretation. Long-term remission, relapse, functional recovery, and adherence could not be systematically evaluated. Finally, although we used independent dual coding and an explicit positionality statement (Sections 3.5–3.6), the analytic team’s clinical involvement and shared commitment to recognizing functional neurological disorder may have introduced interpretive bias toward a neuropsychiatric formulation; readers should weigh this when interpreting the themes.

Three further limitations bear on the strength of any inference. First, this was an uncontrolled series with no comparison group and only short-term follow-up; the observed reductions in episode frequency are descriptive and cannot establish treatment efficacy or causation. Second, treatment response was recorded as a clinical outcome only and was not used as diagnostic evidence, because improvement with psychological care does not differentiate functional/dissociative seizures from epilepsy or other disorders. Third, beyond the absence of EEG, neurology or epileptology consultation could not be obtained during the assessment period because of cost, distance, and the national shortage of specialist services (Section 2.5); the differential diagnosis could not be completed to the standard achievable in better-resourced settings, and a comorbid or alternative neurological cause cannot be excluded in any individual case. Together with the small, single-center, all-female sample, these constraints define the series as provisional and hypothesis-generating rather than confirmatory. Finally, because the six cases were ascertained within a large, systematically screened cohort (Section 3.2) rather than by dedicated neurological case-finding, the inference that functional/dissociative seizures are under-recognised in this population is grounded in that screening context; the true magnitude of under-recognition, and any generalisation beyond comparable high-adversity female rehabilitation populations, remain to be established in prospective, multi-site work.

## 8 Conclusion

Clinically suspected FDS was identified among young women at a single Ethiopian rehabilitation center and may be under-recognized, misclassified, and undertreated. The six cases suggest that functional seizure-like episodes in this context are best conceptualized as neuropsychiatric, culturally mediated, gendered, and structurally shaped. Making FDS visible in Ethiopia requires culturally adapted screening, trauma-informed assessment, suicide-risk evaluation, management of psychiatric comorbidity, respectful engagement with explanatory models, and referral pathways for settings where video-EEG is unavailable. Addressing this gap represents both a clinical priority and a public health responsibility and provides a necessary foundation for advancing systematic FDS research in Ethiopia. These conclusions derive from a single-center case series and require confirmation in larger, prospective studies with electrophysiological support.

## Data Availability

The datasets presented in this article are not readily available because of ethical restrictions. Requests to access the datasets should be directed to the corresponding author.
